# Road traffic noise exposure and blood DNA methylation at birth and in childhood: An epigenome-wide *meta*-analysis

**DOI:** 10.1016/j.envint.2025.109976

**Published:** 2025-12-02

**Authors:** Zhebin Yu, Irene Fontes Marques, Simon Kebede Merid, Kimberley Burrows, Ana Goncalves Soares, Andrei Pyko, Mikael Ögren, Göran Pershagen, Johanna Lepeule, Norun Hjertager Krog, Gunn Marit Aasvang, Michelle S.W. Kusters, Maria Foraster, Mariona Bustamante, Miriam Leskien, Elisabeth Thiering, Ahmed Elhakeem, Annette Peters, Gerard H. Koppelman, Ulrike Gehring, Judith M. Vonk, Ayoung Jeong, Medea Imboden, Nicole Probst-Hensch, Roel Vermeulen, Mark Nieuwenhuijsen, Mònica Guxens, Marie Standl, Vincent W.J. Jaddoe, Stephanie J London, Erik Melén, Janine F Felix, Olena Gruzieva

**Affiliations:** a Institute of Environmental Medicine, Karolinska Institutet, Stockholm, Sweden; b The Generation R Study Group, Erasmus MC, University Medical Center Rotterdam, Rotterdam, the Netherlands; c Department of Pediatrics, Erasmus MC, University Medical Center Rotterdam, Rotterdam, the Netherlands; d Department of Clinical Science and Education, Södersjukhuset, Karolinska Institutet, Stockholm, Sweden; e Centre for Academic Child Health, Population Health Sciences, Bristol Medical School at the University of Bristol, Bristol, UK; f Population Health Science, Bristol Medical School, University of Bristol, Bristol, UK; g MRC Integrative Epidemiology Unit at the University of Bristol, Bristol, UK; h Centre for Occupational and Environmental Medicine, Region Stockholm, Sweden; i Occupational and Environmental Medicine, School of Public Health and Community Medicine, Institute of Medicine, Sahlgrenska Academy, University of Gothenburg, Sweden; j Université Grenoble Alpes, INSERM U1209, CNRS UMR 5309, Institut pour l’Avancée des Biosciences (IAB), Team of Environmental Epidemiology Applied to Development and Respiratory Health, 38000 Grenoble, France; k Department of Air Quality and Noise, Division of Climate and Environmental Health, Norwegian Institute of Public Health, Oslo, Norway; l ISGlobal, Barcelona, Spain; m Universitat Pompeu Fabra, Barcelona, Spain; n Department of Child and Adolescent Psychiatry/Psychology, Erasmus University Medical Centre, Rotterdam, the Netherlands; o PHAGEX Research Group, Blanquerna School of Health Science, Universitat Ramon Llull (URL), Barcelona, Spain; p CIBER Epidemiología y Salud Púbica, Madrid, Spain; q Institute of Epidemiology, Helmholtz Zentrum München- German Research Center for Environmental Health, Neuherberg, Germany; r Institute for Medical Information Processing, Biometry, and Epidemiology, Medical Faculty, Ludwig-Maximilians-Universität München, Munich, Germany; s Department of Pediatrics, Dr. von Hauner Children’s Hospital, LMU University Hospital, Ludwig-Maximilians-Universität München, Munich, Germany; t Chair of Epidemiology, Institute for Medical Information Processing, Biometry and Epidemiology, Médical Faculty, Ludwig-Maximilians-Universität München, Munich, Germany; u Munich Heart Alliance, German Center for Cardiovascular Health (DZHK e.V., partner-site Munich), Munich, Germany; v University of Groningen, University Medical Center Groningen, Beatrix Children’s Hospital, Department of Pediatric Pulmonology and Pediatric Allergology, Groningen, the Netherlands; w University of Groningen, University Medical Center Groningen, GRIAC Research Institute, Groningen, the Netherlands; x Institute for Risk Assessment Sciences, Utrecht University, Utrecht, the Netherlands; y University of Groningen, University Medical Center Groningen, Department of Epidemiology, Groningen, the Netherlands; z Swiss Tropical and Public Health Institute, Allschwil, Switzerland; aa University of Basel, Basel, Switzerland; ab ICREA, Barcelona, Spain; ac German Center for Child and Adolescent Health (DZKJ), Munich, Germany; ad German Center for Lung Research (DZL), Munich, Germany; ae Division of Intramural Research, National Institute of Environmental Health Sciences, National Institutes of Health, Research Triangle Park, NC, USA

**Keywords:** Road traffic noise, DNA methylation, Epigenome-wide association analysis, Birth cohorts

## Abstract

Road traffic noise exposure has been associated with multiple adverse outcomes in epidemiological studies. However, the underlying biological mechanisms remain unclear. The aim of this study was to investigate the association between road traffic noise exposure and cord blood and child blood DNA methylation (DNAm).

Data from six European studies (BAMSE, Generation R, HELIX, INMA, LISA, PIAMA) were used to perform the discovery epigenome-wide *meta*-analysis. Prenatal, infancy, and recent road traffic noise exposure was assessed at the residential addresses. Blood DNAm was measured using the Illumina 450 K or EPIC arrays. To identify differentially methylated positions (DMPs), we fitted robust linear regression models for each cohort, and the results were subsequently *meta*-analyzed. Differentially methylated regions (DMRs) were identified using Comb-p and DMRcate. Findings were then looked-up in the independent ALSPAC cohort, in which noise was measured categorically.

A total of 1477 newborns with DNAm data in cord blood, and 1129 and 2065 with DNAm in child blood (age 4–6 and age 8–10 years, respectively) were included in the discovery *meta*-analysis. We did not observe genome-wide significant (False Discovery Rate (FDR) < 0.05) DMPs associated with road traffic noise exposure. However, 46 DMPs reached suggestive significance (P < 1 × 10^–5^) across different time windows. One CpG site (cg09400092, annotated to *SSTR1*) associated with recent noise exposure at age 8–10 years was also significantly associated in the ALSPAC cohort (same direction of association with P = 0.00165). In addition, we identified a total of 93 FDR significant DMRs, of which 14 were nominally significant in the ALSPAC study.

In conclusion, we observed suggestive evidence of an association between road traffic noise exposure and DNAm in child blood. This may indicate that differential DNAm plays a role in the biological mechanism underlying health effects of noise exposure.

## 1. Introduction

Road traffic noise is the second leading environmental stressor in Europe, according to the World Health Organization (WHO) (Basner and McGuire, 2018). A recent assessment of exposure to transportation noise showed that around 20 % of the European population is living in areas with harmful levels of noise (exceeding 55 dB in Day-Evening-Night Level (L_den_)) (Peris, 2020), and the population’s exposure to transportation noise is projected to increase due to urban expansion and growing demand for mobility (Peris, 2020). Numerous epidemiological analyses have demonstrated associations between noise exposure and adverse health effects, best documented for cardiovascular outcomes (Münzel et al., 2024), but also metabolic (Vienneau et al., 2024; Persson et al., 2024; Eze et al., 2017), respiratory (Liu et al., 2021; Eze et al., 2018), and reproductive outcomes (Sørensen et al., 2024) among adults, as well as hearing function (Selander et al., 2016), cognitive, behavioral, learning, and other neurodevelopmental outcomes among children and adolescents (Roche et al., 2024; Terzakis et al., 2022). Children may be more susceptible to the effects of noise due to the developing auditory system and because the impact of noise on hearing and quality of life at early stages of development can influence child health trajectories (Balk et al., 2023).

The proposed pathophysiological mechanisms underlying the adverse effect of noise exposure relate to the activation of stress response pathways (Münzel et al., 2017; Kuntic et al., 2023) and nighttime sleep disturbance (Kröller-Schön et al., 2018) (with dysregulation of the circadian clock), supported by animal studies (Münzel et al., 2021) and gene-environment studies (Eze et al., 2017). Noise can activate downstream stress response, such as the activation of the sympathetic nervous system (SNS) and the hypothalamic–pituitary–adrenal (HPA) axis, which further converges in oxidative stress and inflammation associated with endothelial Nitric Oxide Synthase (eNOS) uncoupling, endothelial dysfunction, high blood pressure and hyperglycemia, subsequently triggering adverse health outcomes (Sørensen et al., 2024).

DNA methylation (DNAm), a process during which methyl groups are added to the C5 position of the cytosine within a cytosine-guanine (CpG) dinucleotide, is the most widely studied epigenetic mark and may be a potential mechanism through which noise exposure may impact health outcomes (Leso et al., 2020). Indeed, noise exposure has been reported to be associated with differential DNAm at specific genes in rat brain tissue (Guo et al., 2017). Yet evidence of associations between road traffic noise exposure and DNAm in humans is scarce. The Swiss SAPALDIA cohort (Study on Air Pollution And Lung Disease In Adults) reported that long-term exposure to source-specific (road traffic, aircraft, railway) noise in adults was associated independently of exposure to traffic-related air pollution with differential blood DNAm of genes annotated to pathways related to inflammation, cellular development, and immune response (Eze et al., 2020). In a subset of the Isle of Wight birth cohort (Commodore et al., 2019), self-reported frequencies of heavy vehicles driving by the residential address was found to be associated with blood DNAm at 34 CpG sites at age 18 years. Another study, conducted among 610 female participants in Sweden (318 breast cancer cases and 292 controls), showed that road traffic noise exposure was associated with blood DNAm in two core circadian genes (Thacher et al., 2024). To the best of our knowledge, no epigenome-wide association study has been conducted in relation to noise exposure among children.

In the current study, we aimed to investigate the association between prenatal road traffic noise exposure and cord blood DNAm, as well as between infancy and recent road traffic noise exposure and child blood DNAm in a multi-cohort epigenome-wide association *meta*-analysis.

## 2. Methods

### 2.1. Study population

A total of six European birth cohorts in the Pregnancy And Childhood Epigenetics Consortium (PACE) (Felix et al., 2018) were included in the discovery *meta*-analysis: BAMSE (Children, Allergy, Environment, Stockholm, Epidemiology), the Generation R Study, HELIX (Human Early Life Exposome), INMA (Environment and Childhood), LISA (Influence of Life-style factors on Development of the Immune System and Allergies in East and West Germany) (Heinrich et al., 2002) and PIAMA (Prevention and Incidence of Asthma and Mite Allergy). BAMSE comprises two different DNAm datasets from two separate projects, i.e., Mechanisms of the Development of Allergy that was assessed for genome-wide DNAm together with the PIAMA study (MeDALL) and EpiGene (Xu et al., 2018), while HELIX comprises one DNAm dataset including data from six independent European birth cohorts: BiB (Born in Bradford), EDEN (Study of determinants of pre- and postnatal development), INMA, KANC (Kaunas Cohort), MoBa (The Norwegian Mother and Child Cohort Study), Rhea (Mother-Child Cohort in Crete).

Detailed information about each cohort including recruitment and eligibility is provided in the Supplemental methods. Ethical approval for each cohort was granted by local institutional review boards and informed consent was obtained.

### 2.2. Noise exposure assessment

Long-term exposure to road traffic noise was estimated at participants’ residential addresses. Road traffic noise exposure levels are expressed in L_den_ based on penalties of noise exposure during evenings (by 5 dB) and nighttime (by 10 dB). Noise levels were modeled using standardized approaches, incorporating traffic flow, road characteristics, land use, and building geometry. Detailed descriptions of noise exposure assessment at each cohort are provided in the Supplemental Methods. For the cord blood analyses, we calculated prenatal road traffic noise exposure as the average noise levels during pregnancy at the maternal residential addresses. For the child blood analyses, we calculated infancy road traffic noise exposure as the average noise levels from birth up to the child’s first birthday, and also the recent road traffic noise exposure (in early and late childhood) as the average noise levels for the 12 months before the biosampling date. Changing address during the exposure period was taken into account by using time-weighted averages across all residential histories.

### 2.3. DNA methylation profiling

DNAm was measured in cord blood or child blood using the Illumina Infinium 450 K array in all included cohorts except LISA, where the EPIC array (version 1) was used. Sampling processing, quality control and normalization were handled by each cohort with details presented in the Supplemental Methods. Untransformed DNAm beta values were used as the outcome ranging from 0 to 1, with higher values indicating higher level of methylation at the CpG site.

### 2.4. Statistical analysis

**Fig. 1.**
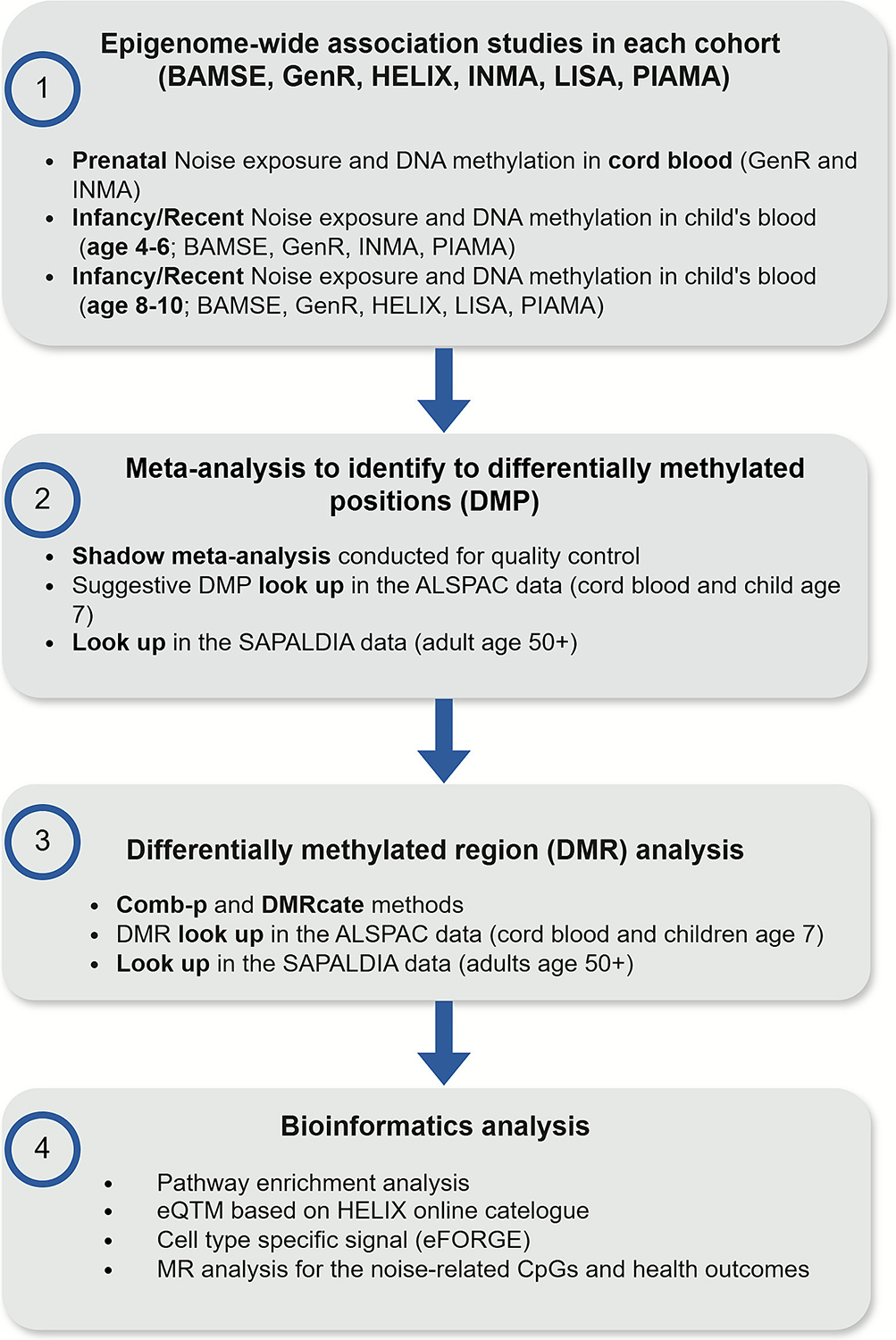
Summary of the study design.

The overall design of the study is presented in Fig. 1.

### 2.5. Cohort-specific analysis

In the discovery phase, six cohorts (BAMSE, Generation R, HELIX, INMA, LISA, PIAMA) conducted the EWAS analyses locally following a prespecified analysis plan and common statistical code. Associations between noise exposure and methylation levels across the epigenome were assessed using multiple robust linear regression analyses for each CpG site individually, implemented with the limma R package. Road traffic noise exposure was entered into the model as continuous variable in dB. Effect estimates are reported per 10 dB increase in noise levels. Analyses in cord blood were adjusted for an *a priori* selected panel of covariates: child sex, maternal education and cord blood cellular composition (Natural killer cells, B cells, CD4T and CD8T lymphocytes, Monocytes, Granulocytes, Nucleated red blood cells) (Gervin et al., 2019); while analyses in children were adjusted for child sex, maternal education, child age at biosampling, and blood cellular composition (Natural killer cells, B cells, CD4T and CD8T lymphocytes, Monocytes, Granulocytes) (Houseman et al., 2012). Information on cohort-specific data collection of the covariates as well as association between noise and cell types are presented in detail in Supplemental methods. Because several cohort-specific EWAS results showed potential inflation (lambda values > 1.2), we applied the Bacon (van Iterson et al., 2017) method to all cohort-specific EWAS. The lambda values for cohort-specific EWAS results before and after Bacon are presented in Supplemental Table S1. A sensitivity analysis was conducted by further adjusting maternal smoking during pregnancy in cord blood analysis, and for both maternal smoking during pregnancy and environmental tobacco exposure in child blood analyses. We also ran analyses further adjusting for ambient air pollution (particular matter ≤ 2.5 μm (PM_2.5_) and Black carbon) exposure modelled for the same exposure time-windows as noise exposure in five of the included cohorts (BAMSE Epigene,PIAMA, Generation R, LISA and HELIX). Details for PM_2.5_ and black carbon exposure assessment can be found elsewhere (Yu et al., 2024; Eeftens et al., 2012).

### 2.6. Meta-analysis for Differentially Methylated Positions (DMP)

The results based on 450 K and EPIC arrays were *meta*-analyzed. The analysis was restricted to probes assessed in both 450 K and EPIC arrays, since only one cohort (LISA) measured DNAm using the EPIC array. We conducted five separate *meta*-analyses, i.e., for prenatal noise exposure and cord blood DNAm, infancy noise exposure and DNAm in early (4–6 years) and late (8–10 years) childhood, as well as recent noise exposure and DNAm in early and late childhood. Cohort-specific EWAS results after Bacon correction were *meta*-analyzed using fixed-effects inverse variance-weighting in METAL (Willer et al., 2010). All *meta*-analyses were independently repeated at Erasmus MC in Rotterdam and results were compared to minimize human error. We filtered out all cross-reactive probes defined by Chen et al (Chen et al., 2013), probes located on the sex chromosomes, and probes only available in one cohort (numbers of probes per model are presented in the Supplemental Methods). P-values were then False Discovery Rate (FDR) corrected for multiple comparisons using the Benjamini-Hochberg procedure (Benjamini and Hochberg, 1995). Genome-wide significance was defined as FDR P-value < 0.05 and suggestive significance as an absolute P-value < 1 × 10^–5^. We calculated the I^2^ statistic to explore heterogeneity across cohorts (Higgins and Thompson, 2002). Leave-one-study-out analyses were also conducted to explore if any of the individual studies were unduly influencing the findings.

### 2.7. Differentially Methylated Region (DMR) analysis

Differentially methylated regions were identified using the Comb-p (Pedersen et al., 2012) and DMRcate (Peters et al., 2015) methods. Comb-p identifies DMRs by aggregating low p-values from neighboring CpG sites within a specific region, while DMRcate detects DMRs using a tunable kernel smoothing approach applied to association signals. Input for both DMR methods were the discovery *meta*-analyzed EWAS results including regression coefficients, standard errors, uncorrected P-values and chromosome positions (for Comb-p only). Detailed input parameters for DMR analysis are presented in Supplemental methods. We defined DMRs as those identified by both methods following multiple-testing correction (Sidak p-value < 0.05 for Comb-p and FDR-adjusted p-value < 0.05 for DMRcate), requiring at least two significant consecutive CpGs within the DMR. The DMRs were further annotated using the rGREAT method (Gu and Hübschmann, 2023).

### 2.8. Look-up in the ALSPAC study and SAPALDIA study

We performed a look-up of the significant and suggestive findings in 589 newborns and 605 7-year-old children from the ALSPAC (Avon Longitudinal Study of Parents and Children) Study (see Supplemental methods for further information). This approach was chosen because the available data in ALSPAC did not allow road traffic noise exposure to be estimated as a continuous variable. Instead, it was estimated as a categorical exposure (L_den_ < 55 dB, 55–59.9 dB, ≥60 dB) and was entered into the model as an ordinal variable (Gonçalves Soares et al., 2024). Other than that, the EWAS analysis followed the same statistical code as in the discovery analysis. We looked up the suggestive DMPs from the discovery EWAS *meta*-analysis in the ALSPAC results. DMPs with p-values less than 0.05 divided by the number of tests as well as a consistent direction of association were considered significant. DMR analyses (Comb-p and DMRcate) were also conducted based on the ALSPAC EWAS results. We examined overlap between the DMRs from the *meta*-analysis and those in ALSPAC. We considered any DMRs that were FDR significant and that overlapped in terms of chromosome position as significant. Additionally, we report overlapping DMRs at the nominally significant level (absolute p-value < 0.05) with at least one nominally significant CpG (Broséus et al., 2024). We further performed a look-up of our top DMPs and DMRs using publicly available EWAS summary statistics from the SAPALDIA adult cohort, which examined transportation noise exposure in relation to DNAm using linear mixed-effect models (Eze et al., 2020).

### 2.9. Bioinformatics analyses

Both suggestive DMPs as well as all the CpGs within the DMRs were used as input in the follow-up bioinformatics analyses. To test whether methylation levels of CpGs were associated with nearby gene expression levels, we looked them up in two publicly available resources: one dataset using 38 cord blood samples (Rojas et al., 2015; Barrett et al., 2013; Rager et al., 2014) and the HELIX Expression Quantitative Trait Methylation (eQTM) catalogue of children’s blood (Ruiz-Arenas et al., 2022). We also conducted enrichment analysis of the CpGs for Gene Ontology (GO) terms and pathways of the Kyoto Encyclopedia of Genes and Genomes (KEGG) and Reactome using the Enrichr website (Kuleshov et al., 2016). Moreover, we searched whether these CpGs have been previously associated with any exposure or health traits using the EWAS catalog (www.ewascatalog.org) and the EWAS Atlas (https://ngdc.cncb.ac.cn/ewas/atlas/index). We also looked up whether any CpGs have potential causal relationships with any disease using the DMRdb database, a disease-centric Mendelian randomization database (Zheng et al., 2024). Finally, eFORGE version 2.0 was used to test for enrichment of tissue-specific DNaseI hypersensitivity regions (Breeze et al., 2019).

## 3. Results

We *meta*-analyzed results from two cohorts with data on DNAm in cord blood (Generation R and INMA, N = 1477), and five cohorts with data on DNAm in child blood (N = 1129 for early childhood with data from BAMSE, Generation R, INMA, LISA, PIAMA; N = 2065 for late childhood with data from BAMSE, Generation R, HELIX, LISA, PIAMA). The description of demographic and lifestyle characteristics of the included participants as well as road traffic noise exposure levels are presented in Table 1 and in Supplemental Table S2. Road traffic noise exposure varied between cohorts: INMA had the highest level of road traffic noise (61.7 ± 6.1 dB in the prenatal exposure window) and cohorts from Netherlands (PIAMA, Generation R) had the lowest level (53.5 ± 4.6 and 54.5 ± 7.9, respectively, in the infancy exposure window). The quantile–quantile plots of *meta*-analyses did not reveal significant inflation in the distribution of observed *p*-values after BACON correction (lambda values ranged from 0.98 to 1.17, Supplemental Fig. S1).

**Fig. 2.**
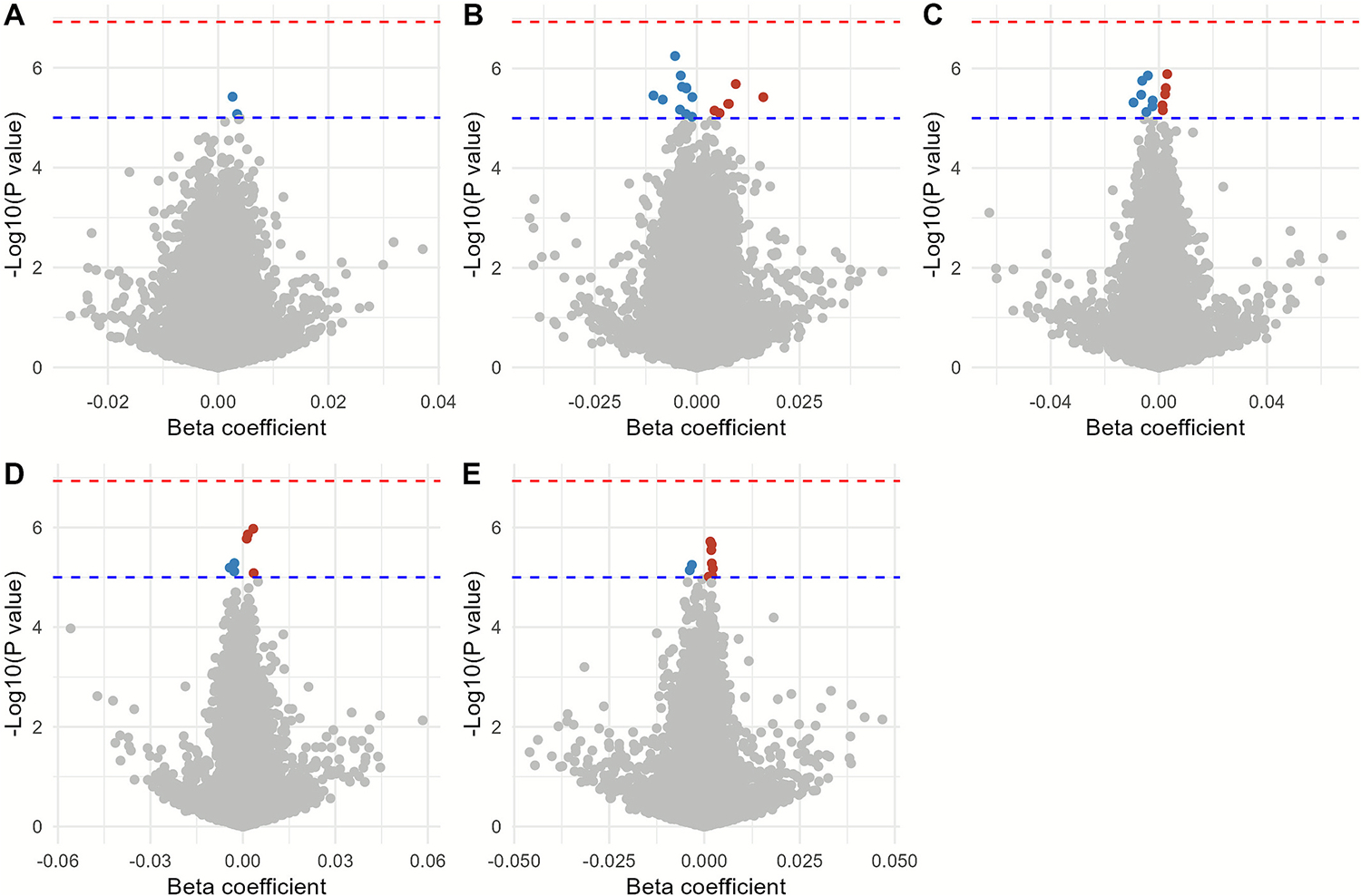
Volcano plots for the meta-analyzed epigenome-wide associations between road traffic noise exposure and DNA methylation in different exposure time windows. (A) Prenatal noise exposure and DNA methylation in cord blood (B) Infancy noise exposure and DNA methylation in children’s blood at 4–6 years; (C) Recent noise exposure and DNA methylation in children’s blood at 4–6 years; (D) Infancy noise exposure and DNA methylation in children’s blood at 8–10 years; (E) Recent noise exposure and DNA methylation in children’s blood at 8–10 years. The x-axis represents the beta coefficient for noise exposure at specific CpG sites (per 10 dB increase), the y-axis represents the −log10 (P values). The blue dashed lines represent the suggestive significant (P value < 10^−5^), while the red dashed line represent the significant after Benjamini–Hochberg correction. The blue dots are suggestive significant CpG sites with negative direction of beta coefficient, the red dots are suggestive significant CpG with positive direction of beta coefficients.

We did not observe genome-wide significant DMPs (FDR < 0.05) in any of the considered exposure time windows. However, 46 DMPs reached suggestive significance (p-values < 1 × 10^–5^) across different exposure periods: two DMPs for prenatal road traffic noise exposure and DNAm in cord blood, 16 and 7 DMPs for infancy road traffic noise exposure and DNAm in early and late childhood, respectively, and 12 and 9 DMPs for recent road traffic noise exposure and DNAm in early and late childhood, respectively (Table 2, Fig. 2). The I^2^ values ranged from 0 to a maximum of 69.6, with 15 CpGs having a value of > 50 (Supplemental Fig. S2). Sensitivity analyses additionally adjusting for maternal smoking during pregnancy and second-hand smoke exposure in childhood did not materially change the coefficients for these DMPs (Supplemental Fig. S3). Leave-one-study-out analyses showed no undue influence from any single cohort (Supplemental Fig. S4), and additional adjustment for ambient PM_2·5_ or black carbon yielded similar estimates with Kolmogorov–Smirnov tests showing no significant differences in the distribution of beta coefficients and p-values (Supplemental Fig. S5). Look up for these DMPs in the EWASs of other exposure time windows suggested stronger consistency of association estimates within the same age groups (Supplemental Fig. S6).

We identified a total of 93 FDR-significant DMRs (88 unique) associated with road traffic noise exposure (overlapping between the Comb-p and DMRcate methods), with 9 for prenatal exposure, 26 and 18 for infancy noise exposure and DNAm in early and late childhood, as well as 12 and 28 for recent noise exposure and DNAm in early and late childhood, respectively). Among these DMRs, 5 were found in two different exposure time windows: chr13:47472050–47472429 annotated to *HTR2A* (infancy exposure with child blood age 4–6 and age 8–10), chr14:95826570–95826997 annotated to *CLMN* (infancy and recent exposure with child blood age 4–6), chr16:787799–788184 annotated to *NARFL* (prenatal exposure with cord blood and infancy exposure with child blood age 4–6), chr4:1041044–1041062 annotated to *FGFRL1* (infancy and recent exposure with child blood age 4–6) and chr6:28583971–28584289 annotated to *SCAND3* (infancy and recent exposure with child blood age 4–6) (Table 3 and Supplemental Table S3).

In the ALSPAC Study, one suggestive CpG in child blood age 8–10 (cg09400092 annotated to *SSTR1*) was significantly associated with recent road traffic noise exposure (same direction of association with p-value = 0.00165). None of the DMRs identified in the discovery *meta*-analysis were associated at the FDR level, but 14 DMRs with at least one overlapping CpG were found to be nominally significant (Table 3 and Supplemental Table S3). In the further look up analysis in the adult cohort (SAPALDIA), we did not replicate the suggestive CpGs. Among the 93 FDR significant DMRs that we identified in children, we found two childhood DMRs were also associated in the SALPADIA study (chr7:27142427–27143586 annotated to *HOXA2* and chr13:47472050–47472429 annotated to *HTR2A*, nominal p-values = 0.013 and 0.024, respectively).

We did not find functional enrichment for the suggestive DMPs and CpGs within the DMRs for GO terms, KEGG or Reactome pathways after FDR correction (Supplemental Table S4–S8). We observed four significant eQTMs for the suggestive DMPs and 135 significant eQTMs for the CpGs within the identified DMRs (Supplemental Table S9). A total of 72 out of these 139 CpG-transcript associations were inverse and 67 were positive. The most statistically significant eQTM was cg26855724 with *CRYZ* expression (log2 fold-change = −0.32;(standard error = 0.25; P value = 3.96E- (Chen et al., 2013). According to the EWAS catalog and EWAS Atlas, the suggestive DMPs and CpGs within the DMR regions have been previously reported in relation to sex, child age, gestational age, pregnancy factors (maternal body mass index, plasma folate, pre-eclampsia), rheumatoid arthritis, mental disorders (attention-deficit/hyperactivity disorder, schizophrenia) and environmental exposures such as ambient air pollution and smoking (Supplemental Table S10). For the Mendelian Randomization analysis (look up in the DMRdb database), we found causal relationships between CpGs (cg00880741 and cg00955808) and multiple diseases such as asthma, metabolic syndrome and sleep disorders (Supplemental Table S11). Finally, the CpGs within the DMRs were found to be enriched in multiple tissues including blood, fetal muscle, lung, and pancreas (Supplemental Figs. S7–S8).

## 4. Discussion

In this epigenome-wide *meta*-analysis, we combined data from six population-based European cohorts to investigate the association between prenatal road traffic noise exposure and DNA methylation in cord blood, as well as infancy and recent road traffic noise exposure and DNA methylation in early and late childhood. Although no differentially methylated positions (DMPs) reached genome-wide significance at any time point, we identified 46 suggestively associated CpG sites, of which one (cg09400092, annotated to *SSTR1*) was also significant in an independent birth cohort (ALSPAC). In addition, we also identified 93 DMRs (88 unique) associated with road traffic noise exposure, of which 14 were also associated at nominal significance in the ALSPAC Study. We did not find significantly enriched biological pathways, but multiple CpGs were associated with gene expression and many were associated with various environmental and lifestyle exposures or health outcomes. Although the identified CpGs may not represent a biological mechanism linking noise exposure to health, they remain of interest as potential markers of road traffic noise exposure.

To our knowledge, this is the first epigenome-wide association study for road traffic noise exposure conducted among children. Differential methylation at one CpG, cg09400092, was associated with road traffic noise exposure in both the *meta*-analysis and the ALSPAC Study. *SSTR1* (Somatostatin Receptor 1) plays a role in several important biological functions including cell growth regulation, hormone regulation and neurotransmission. This gene has been previously reported to be associated with obesity and non-alcoholic fatty liver disease in mice (Huang et al., 2024), a finding in line with the epidemiological associations observed between transportation noise exposure and overweight/obesity (Persson et al., 2024) and diabetes (Eze et al., 2017).

Despite the differences in study design, population, and statistical methods with the previous SAPALDIA study, we observed two overlapping DMRs, annotated to *HOXA2* and *HTR2A*, between the SAPALDIA and the current discovery analysis. The *HOXA2* (homeobox A2) gene encodes a transcription factor that is crucial for embryonic development and is essential for the proper development of facial structures and the middle ear. Mutations in this gene have been linked to microtia and hearing impairment (Brown et al., 2013). This finding may contribute to a better understanding of the mechanisms behind hearing impairment in children following exposure to noise as reported in earlier epidemiological studies (Selander et al., 2016; Balk et al., 2023). The *HTR2A* gene encodes the 5-HT2A receptor, which is a critical component of the serotonergic system in the human brain. One study conducted among 532 Chinese college students (mean age 24.3 years) showed that polymorphisms within the *HTR2A* gene were associated with individual differences in empathic and autistic-like traits (Gong et al., 2015). In a study in mice, stress exposure was associated with expression levels of the *HTR2A* gene (Maple et al., 2015), which aligns with the previous evidence that noise exposure may trigger cortical activation and release of stress hormones (Babisch, 2003), and over time chronic stress may increase risk of cardiovascular and metabolic diseases. Although findings from animal models may not directly translate to humans, they may provide mechanistic insights into how environmental stressors such as road traffic noise exposure could influence gene regulation and may contribute to long-term health effects.

Among the five DMRs that overlapped between exposure windows in the discovery *meta*-analysis, chr16:787799–788184 (annotated to *NARFL*) and chr13:47472050–47472429 (annotated to *HTR2A*) showed significant differential DNAm at different time points: prenatal exposure with cord blood and infancy exposure with child blood age 4–6 for *NARFL*, and infancy exposure with early and late child blood for *HTR2A*, respectively. This finding may indicate that early-life road traffic noise exposure may have persistent effects on these epigenetic patterns from cord blood to child blood, although the current study cannot employ a true longitudinal design due to the limited overlapping samples with DNAm data across ages from the same cohorts. The *NARFL* gene plays a crucial role in cellular defense mechanisms against oxidative stress. For instance, studies showed that deletion or knockdown of this gene in mice can lead to increased levels of reactive oxygen species (ROS), which is in line with the evidence of inflammatory and oxidative downstream effects of noise exposure (Münzel et al., 2017; Daiber et al., 2019).

The strengths of the current study are the relatively large sample size, the objective assessment of road traffic noise exposure, which was based on individual address levels using refined validated models, the availability of epigenome-wide DNAm data in cord blood and child blood, which allowed to investigate the associations of road traffic noise exposure at different exposure time windows, and a harmonized analysis plan.

Our study also has some limitations. Although a p-value threshold of 10^–5^ is commonly used as a suggestive cutoff in EWAS, it is more lenient than conventional FDR-based criteria and may therefore also capture some CpGs that are not truly associated with noise exposure. Therefore, these findings should be considered exploratory rather than genome-wide statistically significant and interpreted with appropriate caution. The included studies generally neither have information on some of the factors affecting noise exposure, such as house characteristics (residential floor, insulation and orientation of the rooms), habits of keeping windows open, nor estimated noise levels on the least exposed facade, which may affect the precision of modelled noise exposure levels and attenuate estimated associations. Noise is a complex exposure with multiple dimensions—such as intensity, duration, frequency spectrum, and individual perception — all of which may influence biological responses through distinct pathways. We used annual average noise exposure (L_den_) to facilitate comparison between pregnancy, 4–6 years, 8–10 years, and potentially adulthood. Although this noise metric is found to be associated with several health outcomes, including noise annoyance, in previous studies (Environmental noise guidelines for the European Region: executive summary, 2025), it does not capture all subjective responses to noise, nor the detailed acoustic characteristics that may drive downstream biological effects. Future studies are warranted to investigate additional aspects of noise exposure, e.g. different lag time, shorter/longer time window, frequency spectrum, noise types and sources, individual perception, exposure reducing behavior, etc. Due to the observational study design, we cannot conclude anything on potential causal relationships. Although we have adjusted for predefined covariates in the main analysis as well as tested the role of maternal smoking and environmental tobacco smoking in the sensitivity analysis, residual confounding e.g, by maternal factors (i.e. maternal stress, maternal nutrition status) cannot be ruled out. Future studies with more comprehensive data on these characteristics are needed to better disentangle the complex relationships. Although low to moderate correlations between road traffic and air pollution were observed (Supplemental Table S12) and additionally adjusted for traffic related air pollution in four study did not materially change the associations of noise with DNAm, further work needs to be done to fully disentangle effects of road traffic noise and traffic related air pollution. Other area-level exposures, such as socioeconomic status (SES) and greenness measured as Normalized Difference Vegetation Index (NDVI) may be also associated with road traffic noise exposure as well as DNAm, and may therefore act as confounder. However, in we examined these correlations in one of the included studies (BAMSE EpiGene) and found very weak correlations between noise exposure and area-level SES (r = 0.02) or NDVI (r = −0.01). Further adjusting for these variables yielded effect estimates that were highly correlated with those from the main model (r = 0.88 for SES-adjusted and r = 0.87 for NDVI-adjusted models), suggesting that these area-level factors are unlikely to have materially confounded our findings. The current study was restricted to the European population due to data availability, which limits the generalization of our findings to other populations. The between-cohort differences in geographical settings, study period, methods for noise exposure assessment, as well as quality control, normalization, and adjustment for technical variation in the DNAm data could to some extent contribute to diluting possible associations, although an earlier published EWAS *meta*-analysis including the same cohorts reported robust results in relation to different data processing methods used across the cohorts for normalization and corrections for technical variables (Joubert et al., 2016). In addition, DNAm signatures are known to be cell- and tissue specific. Like many other EWAS analyses we used cord and peripheral blood cells to investigate the association between environmental exposure and DNAm patterns, which may not be the most directly relevant tissue in the pathway to noise-related health effects. The results from the eFORGE analysis showed enrichment in specific cells and tissues, indicating that future research using other relevant cells and tissues may be useful (Broséus et al., 2024; Mortillo and Marsit, 2023).

In conclusion, we observed suggestive evidence of the association between road traffic noise exposure and DNA methylation in cord and child blood, that overlapped in part with associations observed in adults. Further studies, ideally with larger sample sizes and more specific measures of noise exposure, are needed to provide additional insights into the role of DNA methylation as a marker or underlying mechanism linking road traffic noise exposure to adverse health outcomes.

## Supplementary Material

Supplementary methods

Supplementary tables

Supplementary figures

## Figures and Tables

**Table 1:** Basic characteristics of the cohorts included in the discovery EWAS *meta*-analysis.

Cohorts, Country	Sample size	Female, n (%)	Low/medium maternal education*, n (%)	Maternal smoking during pregnancy, n (%)	Mean (SD) Noise exposure, Lden (dB)			PM2.5 exposure, μg/m3		Black carbon exposure, μg/m3	
					Prenatal	Infancy	Recent	Infancy	Recent	Infancy	Recent
Methylation in the cord blood (N = 1477)											
Generation R, Netherlands	1129	560 (49.6)	447 (39.6)	152 (13.5)	54.4 (7.9)	–	–	–	–	–	–
INMA, Spain	348	178 (51.1)	236 (67.8)	49 (14.1)	61.7 (6.1)	–	–	–	–	–	–
Methylation in the children’s blood (4–6 years, N = 1129)											
BAMSE (MeDALL), Sweden	255	117 (45.9)	126 (49.4)	29 (11.4)	–	58.3 (9.2)	54.6 (11.3)	–	–	–	–
Generation R, Netherlands	371	190 (51.2)	155 (41.8)	34 (9.2)	–	54.5 (7.9)	52.8 (7.4)	–	–	–	–
INMA, Spain	177	93 (52.5)	120 (67.8)	21 (11.9)	–	61.5 (6.5)	61.2 (6.3)	–	–	–	–
LISA, Germany	119	68 (57.1)	37 (31.1)	8 (6.7)	–	59.2 (11.1)	–	–	–	–	–
PIAMA, Netherlands	207	105 (50.7)	132 (62.7)	26 (12.6)		53.5 (4.6)	53.1 (4.0)	–	–	–	–
Methylation in the children’s blood (8–10 years, N = 2065)											
BAMSE (Epigene), Sweden	375	172 (45.9)	188 (50.1)	42 (11.2)	–	58.3 (9.0)	54.7 (10.0)	8.79 (0.88)	8.19 (0.80)	0.98 (0.40)	0.83 (0.34)
BAMSE (MeDALL), Sweden	264	121 (45.8)	128 (48.5)	31 (11.7)	–	58.4 (9.3)	54.9 (10.1)	8.91 (0.98)	8.22 (0.94)	1.02 (0.50)	0.86 (0.43)
Generation R, Netherlands	371	184 (49.6)	147 (39.6)	38 (10.2)	–	55.0 (7.8)	52.5 (7.0)	18.58 (1.36)	14.42 (1.15)	1.72 (0.34)	1.45 (0.30)
HELIX, UK/Spain/Norway/Greece/Lithuania	762	419 (55.0)	347 (45.5)	113 (14.8)	–	57.7 (7.3)	56.9 (8.1)	13.37 (2.59)	13.61 (4.03)	–	–
LISA, Germany	86	49 (57.0)	28 (32.6)	7 (8.1)	–	–	53.2 (7.4)	–	13.85 (0.63)	–	–
PIAMA, Netherlands	207	104 (50.2)	132 (62.7)	25 (12.1)	–	53.5 (4.6)	53.5 (4.2)	16.34 (0.71)	16.35 (0.68)	1.25 (0.27)	1.23 (0.24)

*: Maternal education was classified into lower, middle, and higher. The number and percentage presented refers to the sum of the low and middle categories. The number and percentage presented for maternal smoking refers to those who smoked.−: data were not available for the specific analysis.

**Table 2:** Suggestive Differentially methylated positions (DMPs) related to road traffic noise exposure in the discovery *meta*-analysis.

Exposure	Methylation	CpG site	Chromosome	Location	Annotated Gene	Effect*	SE	P value	Direction	I^2^
Prenatal	Cord blood	cg26632494	chr5	64,920,945	*C5orf44*	0.0030	1.00E-04	3.80E-06	++	4.262
	Cord blood	cg15963326	chr19	50,037,085	*RCN3*	0.0030	1.00E-04	8.47E-06	++	0.535
Infancy	Children age 4–6	cg09216236	chr1	65,719,627	*DNAJC6*	−0.0053	1.06E-04	5.64E-07	−?—	37.2
		cg16494747	chr16	82,660,206	*CDH13*	−0.0039	8.17E-05	1.39E-06	−——	50.8
		cg00880741	chr2	3,750,539	*DCDC2C*	0.0094	1.97E-04	2.06E-06	++-++	22.6
		cg14463465	chr10	67,387,893		−0.0037	7.74E-05	2.34E-06	−——	68.4
		cg18627179	chr19	17,970,627	*RPL18AP3*	−0.0026	5.49E-05	2.44E-06	?-?-?	61.8
		cg16766966	chr7	75,610,520	*POR*	−0.0026	5.48E-05	2.53E-06	−——	0
		cg21984532	chr7	130,789,927	*FLJ43663*	−0.0106	2.29E-04	3.50E-06	−???-	0
		cg21254083	chr11	119,598,909	*PVRL1*	−0.0011	2.41E-05	3.76E-06	+——	50.8
		cg26168643	chr13	88,328,009	*SLITRK5*	0.0161	3.48E-04	3.77E-06	++-++	0
		cg00955808	chr6	21,754,525	*FLJ22536*	−0.0084	1.82E-04	4.23E-06	−——	0
		cg15352671	chr11	1,331,497	*LOC255512*	0.0077	1.69E-04	5.14E-06	++++-	63.5
		cg07730360	chr3	128,845,626	*RAB43*	−0.0041	9.17E-05	6.72E-06	−——	59.6
		cg04442638	chr17	73,873,823	*TRIM47*	0.0043	9.62E-05	7.04E-06	+++++	0
		cg11573854	chr7	1,250,964	*UNCX*	0.0056	1.25E-04	7.91E-06	++-+-	67.3
		cg05126036	chr1	155,920,658	*ARHGEF2*	−0.0027	5.97E-05	8.23E-06	−——	0
		cg22506548	chr1	2,996,949	*PRDM16*	−0.0012	2.64E-05	9.36E-06	−+—	3.5
	Children age 8–10	cg07056962	chr6	99,962,757	*USP45*	0.0033	6.86E-05	1.06E-06	+++++	0
		cg06819431	chr1	153,605,367	*C1orf77*	0.0016	3.37E-05	1.38E-06	++++-	0
		cg06918898	chr2	208,576,002	*CCNYL1*	0.0013	2.64E-05	1.68E-06	+++++	42.6
		cg08991927	chr5	146,436,805	*PPP2R2B*	−0.0028	6.08E-05	5.21E-06	−——	0
		cg07207652	chr17	45,798,257	*TBX21*	−0.0043	9.46E-05	6.42E-06	−——	0
		cg24949488	chr10	98,064,362	*DNTT*	−0.0028	6.26E-05	7.62E-06	−——	6
		cg21874312	chr12	131,687,539	*LOC116437*	0.0035	7.82E-05	8.25E-06	+++++	0
Recent	Children age 4–6	cg09294589	chr3	33,155,133	*CRTAP*	0.0031	6.38E-05	1.30E-06	++++	0
		cg03062717	chr19	35,940,483	*FFAR2*	−0.0041	8.43E-05	1.40E-06	−—	68.1
		cg14948804	chr4	70,896,083	*HTN3*	−0.0062	1.29E-04	1.78E-06	−—	0
		cg09418519	chr4	15,683,124	*LOC285550*	0.0026	5.45E-05	2.49E-06	++++	0
		cg08344081	chr6	41,339,785	*NCR2*	0.0023	4.97E-05	3.31E-06	++++	65.4
		cg19841572	chr12	2,039,752	*CACNA2D4*	−0.0065	1.41E-04	3.39E-06	−—	0
		cg03825158	chr16	49,612,801	*ZNF423*	−0.0023	5.04E-05	4.43E-06	−-+-	68.7
		cg10825234	chr4	1,041,061	*FGFRL1*	−0.0094	2.05E-04	4.85E-06	−–+	52.8
		cg12639781	chr10	180,504	*ZMYND11*	0.0013	2.87E-05	5.52E-06	++++	69.6
		cg12281527	chr8	142,200,092	*DENND3*	−0.0024	5.26E-05	5.78E-06	−-+-	4.9
		cg23281714	chr1	24,829,351	*RCAN3*	0.0014	3.22E-05	6.94E-06	+++-	24.9
		cg26397250	chr4	642,765	*PDE6B*	−0.0046	1.03E-04	7.52E-06	−-+-	19.9
	Children age 8–10	cg21754727	chr3	47,883,023	*DHX30*	0.0016	3.39E-05	1.91E-06	++++++	15.6
		**cg09400092**	**chr14**	**38,677,295**	***SSTR1***	**0.00120**	**4.14E-05**	**2.19E-06**	**++++?-**	**16.6**
		cg13150785	chr15	41,138,160	*SPINT1*	0.0019	3.96E-05	2.84E-06	++-+++	56.9
		cg00680551	chr8	103,135,595	*NCALD*	0.0020	4.38E-05	5.24E-06	+++++-	0
		cg14166948	chr1	1,099,563	*TTLL10*	−0.0033	7.16E-05	5.64E-06	−—?+	63.1
		cg12117616	chr8	42,546,842	*CHRNB3*	0.0023	5.02E-05	6.67E-06	++++++	62.6
		cg17063840	chr15	40,649,930	*DISP2*	−0.0038	8.50E-05	7.19E-06	−———	22.7
		cg14144314	chr14	101,506,379	*MIR376B*	0.0021	4.72E-05	9.28E-06	+++++-	0
		cg20537325	chr10	131,348,421	*MGMT*	0.0012	2.69E-05	9.68E-06	++++++	4.2

*: Effect estimates presented as per 10 dB increase in the road traffic noise exposure. SE: standard error.

Direction: direction of effect across cohorts included in the statistical model (Generation R and INMA for prenatal & cord blood; BAMSE_MEDALL, Generation R, PIAMA, INMA, LISA for infancy&children age 4–6; BAMSE Epigene, BAMSE MEDALL, Generation R, HELIX, PIAMA for infancy & children 8–10; BAMSE MEDALL, Generation R, PIAMA, INMA for recent&children age 4–6; BAMSE Epigene, BAMSE MEDALL, Generation R, HELIX, PIAMA, LISA for recent & children 8–10): Higher road traffic noise n exposure was associated with higher (+) or lower (−) DNA methylation, or missing (?) result. Cohort-specific estimates can be found in Supplemental Fig. 2.

cg09400092, in bold, was also associated in the ALSPAC data with same direction of association and P value 0.00165.

**Table 3:** Differentially methylated regions (DMRs) related to road traffic noise exposure.

Exposure time window	Methylation	Results from Combp method			Results from the DMRcate method				Annotated Gene
		DMRs	N of CpGs	Sidak *P*-value	DMRs	N of CpGs	FDR *P* value	Meandiff*	
Infancy	Children age 4–6	chr1:3036168–3036359	2	3.67E-02	chr1:3036168–3036358	2	8.00E-03	−2.27E-04	*PRDM16*
		chr13:88328009–88329681	8	8.83E-23	chr13:88328009–88330200	11	1.90E-14	7.84E-04	*SLITRK5*
		chr22:45809244–45810044	16	1.04E-11	chr22:45808669–45810043	17	8.49E-13	3.73E-04	*RIBC2*
	Children age 8–10	chr16:2907695–2908246	7	4.74E-06	chr16:2907517–2908934	12	9.78E-06	5.08E-04	*PRSS22*
		chr17:55962522–55963103	5	2.40E-08	chr17:55962155–55963458	7	1.61E-06	−3.60E-04	*MRPS23*
		chr22:45809319–45809794	13	1.96E-04	chr22:45809244–45810043	16	1.73E-03	2.14E-04	*RIBC2*
		chr6:31691426–31691813	14	1.38E-02	chr6:31691227–31692026	21	4.20E-03	1.77E-04	*C6orf25*
		chr7:27142427–27143586	20	4.18E-11	chr7:27142100–27143806	27	3.10E-10	−2.69E-04	*HOXA2*
Recent	Children age 4–6	chr4:187422005–187422120	5	3.68E-03	chr4:187422005–187422119	5	1.42E-02	6.84E-04	*MTNR1A*
		chr6:8436218–8436467	4	1.84E-02	chr6:8435883–8436466	10	5.50E-03	2.70E-04	*SLC35B3*
	Children age 8–10	chr11:73357019–73357397	8	8.71E-04	chr11:73356744–73357741	10	4.21E-04	−1.59E-04	*PLEKHB1*
		chr21:47717864–47718081	3	1.62E-02	chr21:47717406–47718080	5	6.52E-03	−2.04E-04	*YBEY*
		chr6:70990226–70990362	3	3.58E-02	chr6:70990226–70990361	3	1.35E-02	−3.37E-04	*COL9A1*
		chr9:34370781–34370895	4	2.74E-03	chr9:34370781–34371380	5	2.25E-03	−4.29E-04	*KIAA1161*

Only DMRs validated in the ALSPAC data were presented here. Full list of DMRs identified in the discovery *meta*-analysis can be found in Supplemental Table 3.

*: Mean coefficients of all the CpGs within the region.

## Data Availability

The analysis plan and common statistical code are available at the online repository: https://github.com/kevininef/NoiseEWAS. The full EWAS *meta*-analysis results can be found at: https://zenodo.org/records/16911597. Access to cohort specific data is possible by contacting each one of the cohorts.

## Appendix A. Supplementary data

Supplementary data to this article can be found online at https://doi.org/10.1016/j.envint.2025.109976.
