# Supplementary material for: Road traffic noise exposure and blood DNA methylation at birth and in childhood: An epigenome-wide meta-analysis
Source: Environ Int. Author manuscript; Available in PMC 2026 Jan 23. (PMC12829588; doi:10.1016/j.envint.2025.109976)
Supplement: Supplementary methods [file NIHMS2138169-supplement-Supplementary_methods.docx]

**Road traffic noise exposure and blood DNA methylation at birth and in childhood: An epigenome-wide meta-analysis**

Supplemental Methods

[ALSPAC 2](#_Toc213576259)

[BAMSE 3](#_Toc213576260)

[Generation R 6](#_Toc213576261)

[HELIX 9](#_Toc213576262)

[INMA 11](#_Toc213576263)

[LISA 14](#_Toc213576264)

[PIAMA 16](#_Toc213576265)

[Differentially methylated region (DMR) analysis input parameters 19](#_Toc213576266)

[Table 1 Number of probes removed during the quality control for the meta-analysis 20](#_Toc213576267)

[Associations between road traffic noise exposure and cell types estimated using the Houseman method 22](#_Toc213576268)

[Comparison for the beta coefficients derived from the EWAS models adjusting for 6 cell types (Houseman) and 12 cell types (Salas) 22](#_Toc213576269)

### ALSPAC

**Design and Study population**

Pregnant women resident in Avon, UK with expected dates of delivery between 1st April 1991 and 31st December 1992 were invited to take part in the study (1,2). 20,248 pregnancies have been identified as being eligible and the initial number of pregnancies enrolled was 14,541. Of the initial pregnancies, there was a total of 14,676 fetuses, resulting in 14,062 live births and 13,988 children who were alive at 1 year of age. When the oldest children were approximately 7 years of age, an attempt was made to bolster the initial sample with eligible cases who had failed to join the study originally. As a result, when considering variables collected from the age of seven onwards there are data available for more than the 14,541 pregnancies mentioned above. The total sample size for analyses using any data collected after the age of seven is therefore 15,447 pregnancies, resulting in 15,658 fetuses. Of these 14,901 children were alive at 1 year of age.

Please note that the study website contains details of all the data that is available through a fully searchable data dictionary and variable search tool: http://www.bristol.ac.uk/alspac/researchers/our-data/.

Ethical approval for the study was obtained from the ALSPAC Ethics and Law Committee and the Local Research Ethics Committees. Consent for biological samples has been collected in accordance with the Human Tissue Act (2004). Informed consent for the use of data collected via questionnaires and clinics was obtained from participants following the recommendations of the ALSPAC Ethics and Law Committee at the time.

**Road traffic noise exposure assessment**

Noise exposure was based on road traffic noise maps from the UK Department of Environment, Food and Rural Affairs (DEFRA, 2006), which were generated under EC Directive 2002/49/EC (Assessment and Management of Environmental Noise) in the framework of the European Noise Directive (END). The primary noise indicators were Lden and Lnight, and these were obtained by doing an intersection between noise maps and geocodes. Lden is the long-term average indicator designed to assess annoyance and defined by the END. It refers to an annual average of day, evening and night period of exposure. Lnight is the long-term average indicator designed to assess sleep disturbance and defined by the END. It refers to an annual average of night period of exposure. Noise data was only available in categories and was linked to the geocoded addresses of the participants.

**Biological sample collection, DNA methylation quantification, quality control and normalization**

DNA methylation was measured from purified DNA in 1,018 mother-child pairs as part of the Accessible Resource for Integrated Epigenomic Studies (ARIES) sub-study (3). Briefly, DNA was extracted using standard protocol and was bisulfite-converted using the Zymo EZ DNA Methylation^TM^ kit (Zymo, Irvine, CA). DNA methylation was then measured using the Infinium HM450 BeadChip assay (Illumina Inc, San Diego, CA), according to the standard protocol. Initial quality control was performed using GenomeStudio (version 2011.1). Raw IDAT files were preprocessed and normalized using the meffil package (4). Dye bias and background correction were performed using the ‘noob’ method (5), while normalization was performed using functional normalization (6). Batch effects were corrected using principal components calculated from control probes (4) and by regressing out slide on the raw betas before normalization and on the control matrix. In addition, samples with > 10% of CpG sites with a detection p-value >0.01 or a bead count <3 in >10% of probes were removed. DNA methylation values (β-value) were generated for the remaining CpG loci, ranging between 0.0 (unmethylated) to 1.0 (completely methylated).

**Acknowledgements and Funding**

We are extremely grateful to all the families who took part in this study, the midwives for their help in recruiting them, and the whole ALSPAC team, which includes interviewers, computer and laboratory technicians, clerical workers, research scientists, volunteers, managers, receptionists and nurses.

The UK Medical Research Council and Wellcome (Grant ref: 217065/Z/19/Z) and the University of Bristol provide core support for ALSPAC. This publication is the work of the authors and Ana Goncalves Soares and Kimberly Burrows will serve as guarantors for the contents of this paper related to ALSPAC.

A comprehensive list of grants funding is available on the ALSPAC website (http://www.bristol.ac.uk/alspac/external/documents/grant-acknowledgements.pdf); This research was specifically funded by the UK Medical Research Council (MC_UU_12013/1, MC_UU_12013/2 and MC_UU_12013/8), BBSRC (BBI025751/1 and BB/I025263/1), Wellcome (WT092830/Z/10/Z) and LLHW via MRC (G1001357). AGS, AE, and NJT work in a Unit that is funded by the UK Medical Research Council (MC_UU_00011/1&6) and the University of Bristol. This project received funding from the European Union’s Horizon 2020 research and innovation programme (874739 LongITools and 733206 LifeCycle).

**References**

1. Boyd A, Golding J, Macleod J, Lawlor DA, Fraser A, Henderson J, Molloy L, Ness A, Ring S, Davey Smith G. Cohort Profile: The ‘Children of the 90s’; the index offspring of The Avon Longitudinal Study of Parents and Children (ALSPAC). International Journal of Epidemiology 2013; 42: 111-127.
2. Fraser A, Macdonald-Wallis C, Tilling K, Boyd A, Golding J, Davey Smith G, Henderson J, Macleod J, Molloy L, Ness A, Ring S, Nelson SM, Lawlor DA. Cohort Profile: The Avon Longitudinal Study of Parents and Children: ALSPAC mothers cohort. International Journal of Epidemiology 2013; 42:97-110.
3. Relton CL, Gaunt T, McArdle W, Ho K, Duggirala A, Shihab H, et al. Data Resource Profile: Accessible Resource for Integrated Epigenomic Studies (ARIES). Int J Epidemiol. 2015 May 19;dyv072.
4. Min JL, Hemani G, Davey Smith G, Relton C, Suderman M. Meffil: efficient normalization and analysis of very large DNA methylation datasets. Bioinformatics. 2018 Dec 1;34(23):3983–9.
5. Triche TJ, Weisenberger DJ, Van Den Berg D, Laird PW, Siegmund KD. Low-level processing of Illumina Infinium DNA Methylation BeadArrays. Nucleic Acids Res. 2013 Apr;41(7):e90.
6. Fortin JP, Labbe A, Lemire M, Zanke BW, Hudson TJ, Fertig EJ, et al. Functional normalization of 450k methylation array data improves replication in large cancer studies. Genome Biol. 2014 Dec 3;15(12):503.

### BAMSE

**Design and Study population**

BAMSE (Children, Allergy, Milieu, Stockholm, Epidemiology in Swedish) is a prospective population-based cohort study of children recruited at birth and followed during childhood. Details of the study design, inclusion criteria, enrolment and data collection are described elsewhere(1). In short, 4,089 children born between 1994 and 1996 in four municipalities of Stockholm County were enrolled. At baseline, when the infant was approximately 2 months of age, parents completed a questionnaire that assessed residential characteristics, as well as socioeconomic and lifestyle factors. When children were 1, 2, 4, 8, the parents completed questionnaires focusing on children’s symptoms related to wheezing and allergic diseases, as well as various exposures. The survey response rates were 96%, 94%, 91% and 84%, respectively. Furthermore, blood was obtained at ages 4 and 8 from 2,605 (63.7%) and 2,470 (60.4%) children, respectively. The baseline and follow-up studies were approved by the Regional Ethical Review Board, Karolinska Institutet, Stockholm, Sweden, and the parents of all participating children provided written informed consent

**Road traffic noise exposure assessment**

Traffic noise exposure assessments are performed using data from several national, regional and local authorities. This methodology has been used in several epidemiological studies from our group and extensively validated against European and Nordic methods (9). It is based on a noise database for Stockholm County representing the period from 1990 and onwards. The database includes 3D terrain data as well as information on ground surface, road net, daily traffic flows, speed limits and percentage of heavy vehicles. Noise levels for each residential were calculated as the equivalent continuous A-weighted sound pressure level (LAeq) at the most exposed façade for day (07:00–19:00 h), evening (19:00–22:00 h), and night (22:00–07:00 h), and expressed as Lden, following penalties of 5 dB and 10 dB for noise occurring during the evening and night, respectively.

**Greenness exposure assessment:** Mean residential greenness within a 300 m buffer was estimated using the Normalized Difference Vegetation Index (NDVI) (10). Briefly, using remote sensing technology, the amount of green vegetation was calculated based on the difference between near infra-red light, reflected by the green vegetation, and red light, not reflected by green vegetation. The values of NDVI range from + 1, which corresponds to dense green vegetation, to −1 which corresponds to water. NDVI values were calculated at a resolution of 30 m by 30 m from cloud-free Landsat Thematic Mapper satellite images obtained from the Global Visualisation viewer from the U.S. Geological Survey. NDVI data used in the study were based on satellite images taken during the spring/summer to capture the greatest variation in vegetation levels.

**Area level Socioeconomic Status**: mean income at the small area market statistics level (SAMS) from Statistical Sweden.

**Biological sample collection, DNA methylation quantification, quality control and normalization**

For this methylation study, we used data from the 4-, and 8- follow-up. At 4 years, epigenome-wide DNA methylation was measured in 256 Caucasian children, at 8 years methylation was measured in 472 Caucasian children. 500 ng DNA per sample underwent bisulfite conversion using the EZ-96 DNA Methylation kit (Zymo Research Corporation, Irvine, USA). Samples were plated onto 96-well plates in randomized order. Samples were processed with the Illumina Infinium HumanMethylation450 BeadChip (Illumina Inc., San Diego, USA). Quality control of analyzed samples was performed using standardized criteria. At 4 years blood methylation data were produced in the Genome Analysis Facility of the University Medical Center Groningen (UMCG) in Holland as part of the MeDALL (Mechanisms of the Development of Allergy) project. DNA methylation data were pre-processed by using the minfi R from the original idat files. Samples that did not provide significant methylation signals in more than 10% of probes (detection P>0.01) were excluded from further analysis. Samples were also excluded in cases of low staining efficiency, low single base extension efficiency, low stripping efficiency of DNA from probes after single base extension, poor hybridization performance, poor bisulphite conversion and high negative control probe staining. For 8 years, samples were excluded in case of sample call rate <99%, colour balance >3, low staining efficiency, poor extension efficiency, poor hybridization performance, low stripping efficiency after extension and poor bisulfite conversion. We also applied multidimensional scaling (MDS) plot to evaluate gender outliers based on chromosome X data, that produced two separated clusters for male and female. Samples that did not belong to the distinct cluster were removed. Furthermore, we applied median intensity plot for methylated and unmethylated intensity by using the minfi R package (samples below the 10.5 cutoff were excluded). Applying these criteria resulted in exclusion of 8 (8-year DNA) samples. Probes with a single nucleotide polymorphism in the single base extension site with a frequency of > 10 % at 4 years and of >5% at 8 years were excluded, as were probes with non-optimal binding (non-mapping or mapping multiple times to either the normal or the bisulphite-converted genome), and the probed belonging to chr X and chr Y, resulting in the exclusion of 46,206 and 46,799 probes (4- and 8- year DNA), leaving a total of 439,306 and 438,713 probes, respectively, in the analyses. Furthermore, we implemented “DASEN” recommended from wateRmelon package to do signal correction and normalization.

***Cell type correction***

We used the Reinius-based Houseman method with the estimate CellCounts function in the Minfi package in R to estimate relative proportions of six white blood cell subtypes (CD4+ T-lymphocytes, CD8+ T-lymphocytes, NK (natural killer) cells, B-lymphocytes, monocytes and granulocytes).

***Batch correction***

Batch correction was attained including the significant (permutation p-value< 10^-4^) principal components derived from the 613 negative control probes presented in 450K arrays. After 10.000 permutations 5 PCs were retained and in additional one batch was also accounted for in the models, based on the bisulfite treatment. The beta-values were batch corrected incorporating these 5 PCs and calculating the residuals of the linear model at 4 years. The covariate batch was also accounted for in the models, based on the bisulfite treatment date at 8 years. At 16 years, the empirical Bayes method via ComBat was applied for batch correction based on sample plate and sentrix position using the sva package in R.


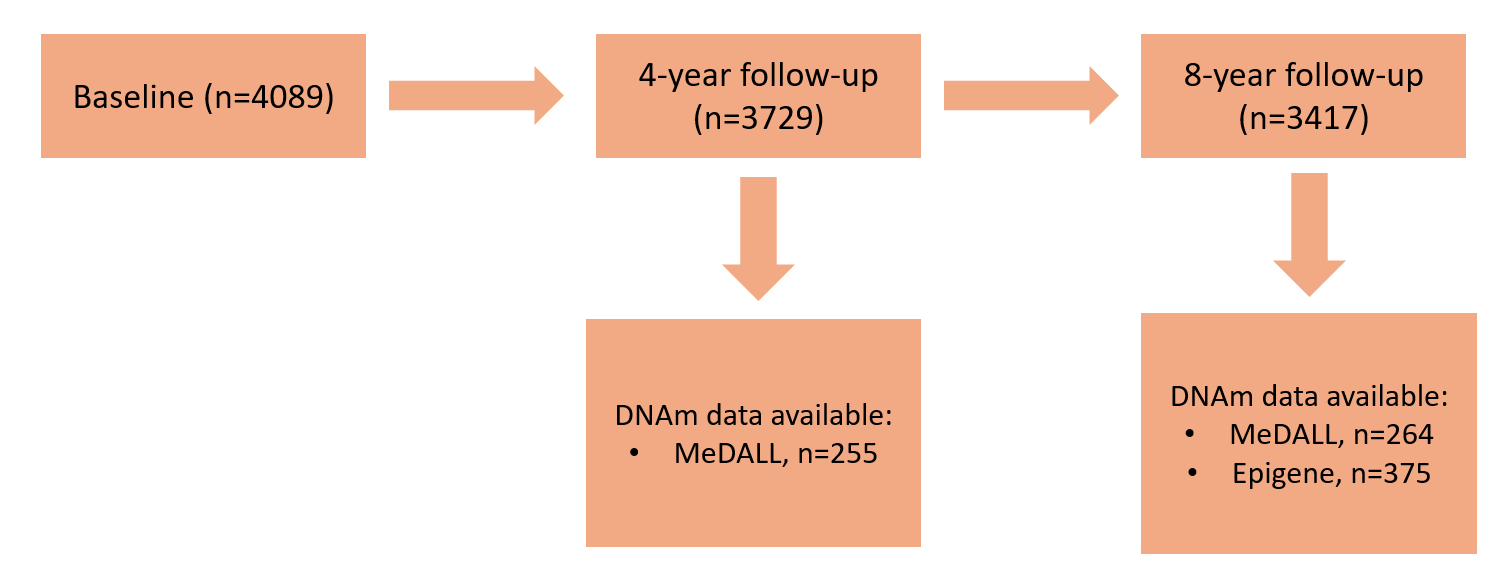


References:

1. Wickman, M., Kull, I., Pershagen, G. & Nordvall, S.L. The BAMSE project: presentation of a prospective longitudinal birth cohort study. *Pediatric allergy and immunology : official publication of the European Society of Pediatric Allergy and Immunology* **13 Suppl 15**, 11-13 (2002)
2. Chen, Y.A., et al. Discovery of cross-reactive probes and polymorphic CpGs in the Illumina Infinium HumanMethylation450 microarray. Epigenetics 8, 203-209 (2013)
3. Xu, C.-J., et al. The emerging landscape of dynamic DNA methylation in early childhood. BMC genomics 18, 25 (2017).
4. Houseman, E.A.*, et al.* DNA methylation arrays as surrogate measures of cell mixture distribution. *BMC bioinformatics* **13**, 86 (2012)
5. Jaffe, A.E. & Irizarry, R.A. Accounting for cellular heterogeneity is critical in epigenome-wide association studies. Genome biology 15, R31 (2014)
6. Pidsley, R., et al. A data-driven approach to preprocessing Illumina 450K methylation array data. BMC genomics 14, 293 (2013)
7. Reinius, L.E., et al. Differential DNA methylation in purified human blood cells: implications for cell lineage and studies on disease susceptibility. PloS one 7, e41361 (2012).
8. Team, R.C. A language and environment for statistical computing. R Foundation for Statistical Computing, Vienna, Austria. ISBN 3-900051-07-0, URL http://www.R-project.org/. (2013).
9. Murzabekov, M, et al. Road-traffic noise exposure and coronary atherosclerosis in the Swedish CArdioPulmonary bioImage Study (SCAPIS). Environ Epidemiol. 2024 Oct 3;8(5):e344.
10. Rhew, IC, et al. Validation of the normalized difference vegetation index as a measure of neighborhood greenness. Ann Epidemiol. 2011 Dec;21(12):946-52.

**Acknowledgements and Funding**

The BAMSE study received funding from the Swedish Research Council (grant no. 2020-01886, 2022-06340), the Swedish Research Council for Health, Working Life and Welfare (FORTE grant no.2017-01146, no.2023-01213), the Swedish Heart-Lung Foundation, Karolinska Institute (no. 2022-01807) and Region Stockholm (ALF project for cohort and database maintenance). We thank the children and parents participating in the BAMSE cohort and all staff involved in the study through the years.

### Generation R

**Design and Study population**

Generation R Study is a population-based prospective birth-cohort from fetal life onwards established in Rotterdam, the Netherlands (1). The Medical Ethical Committee of Erasmus MC, University Medical Center Rotterdam, approved the study (MEC 198.782/2001 /31). Women with an expected delivery date between April 2002 and January 2006 living in Rotterdam were eligible to enroll the study, and written informed consent was obtained from all participants. In 1396 of the 9901 live-born newborns participating in the Generation R Study, we measured genome-wide DNA methylation in cord blood. This subgroup was selected as a relatively homogeneous, European-ancestry subgroup based on genetic information.

**Road traffic noise exposure assessment**

Noise exposure assessment was based on existing European road traffic noise maps, which were generated under EC Directive 2002/49/EC (Assessment and Management of Environmental Noise) in the framework of the European Noise Directive (END) (2). We used noise maps created in 2012 for the municipalities of Rotterdam, Maassluis, Rozenburg, Schiedam, and Vlaardingen in the Netherlands to estimate the outdoor exposure to residential annual average levels of environmental noise, which corresponds to the cohort study period. Noise was modelled using the standardized Dutch calculation methods (‘Standaard Rekenmethoden’, SRM), including surfaces polygon, buildings, barriers, slope, crossings, roundabouts as well as the corresponding emission sources for each of the specific models (3). The maps were developed to estimate noise levels at a height of 4 meters at the most exposed façade of the residential addresses and these maps were then linked with the participants geocoded addresses. The day-evening-night EU noise indicator (L_DEN_) was used in this project. L_DEN_ represents the A-weighted average sound level over 24-h with penalties for evening (+5 dB) and night (+10 dB), as indicated by the END (2). L_DAY_, L_EVENING_, and L_NIGHT_ were defined as the A-weighted mean sound levels obtained during the day (07:00 to 19:00), the evening (19:00 to 23:00), and night (23:00 to 07:00).

**Biological sample collection, DNA methylation quantification, quality control and normalization**

We used the salting-out method to extract DNA from cord blood samples. Five-hundred nanograms of DNA were bisulfite converted using the EZ-96 DNA Methylation kit (Shallow) (Zymo Research Corporation, Irvine, USA). Samples were processed with the Illumina Infinium HumanMethylation450 BeadChip (Illumina Inc., San Diego, USA). Quality control and normalization were performed using the CPACOR workflow (4). Probes with a detection p ≥ 1E^−16^ were set to missing. Intensity values were quantile normalized. We removed arrays with technical problems, a call rate ≤ 95%, or a mismatch between the expected sex of participant and sex determined by chromosome X and Y probe intensities. Probes on the sex chromosomes were removed before the analyses. We used untransformed beta values as measures of DNA methylation, and the final DNA methylation dataset contained information on 458,563 CpGs.

**Cell type and batch correction**

Blood cell type proportions (CD8+ Tcells, CD4+ Tcells, natural kills cells, B cells, monocytes, granulocytes, and nucleated red blood cells in cord blood, and all these cell types except nucleated red blood cells in child peripheral blood) were estimated based on DNA methylation levels using either a cord blood or a peripheral blood-specific reference panel (5, 6). Batch effects were corrected by including a batch variable in the model (DNA methylation plate).

**Acknowledgements and Funding**

The Generation R Study is conducted by Erasmus MC in close collaboration with the School of Law and Faculty of Social Sciences of the Erasmus University Rotterdam, the Municipal Health Service Rotterdam area, Rotterdam, the Rotterdam Homecare Foundation, Rotterdam, and the Stichting Trombosedienst & Artsenlaboratorium Rijnmond (STAR-MDC), Rotterdam. We gratefully acknowledge the contribution of children and parents, general practitioners, hospitals, midwives, and pharmacies in Rotterdam. The generation and management of the Illumina 450K methylation array data (EWAS data) for the Generation R Study was executed by the Human Genotyping Facility of the Genetic Laboratory of the Department of Internal Medicine, Erasmus MC, and the Netherlands. We thank Mr Michael Verbiest, Ms Mila Jhamai, Ms Sarah Higgins, Mr Marijn Verkerk, and Dr Lisette Stolk for their help in creating the EWAS database. We thank Dr Alexander Teumer for his work on the quality control and normalization scripts.

The general design of the Generation R Study is made possible by financial support from Erasmus MC, University Medical Centre Rotterdam, Erasmus University Rotterdam, the Netherlands Organization for Health Research and Development (ZonMw), the Netherlands Organization for Scientific Research (NWO), the Ministry of Health, Welfare and Sport, and the Ministry of Youth and Families. The EWAS data was funded by a grant from the Netherlands Genomics Initiative (NGI)/Netherlands Organization for Scientific Research (NWO) Netherlands Consortium for Healthy Aging (NCHA; project number 050-060-810), by funds from the Genetic Laboratory of the Department of Internal Medicine, Erasmus MC, University Medical Centre Rotterdam (R01HD068437). The project was supported by funding from the European Union’s Horizon 2020 research and innovation program under grant agreements No 733206 (LifeCycle), 874739 (LongITools) and 874583 (ATHLETE), and from the European Joint Programming Initiative ‘A Healthy Diet for a Healthy Life’ (JPI HDHL, NutriPROGRAM project, ZonMw the Netherlands no. 529051022).


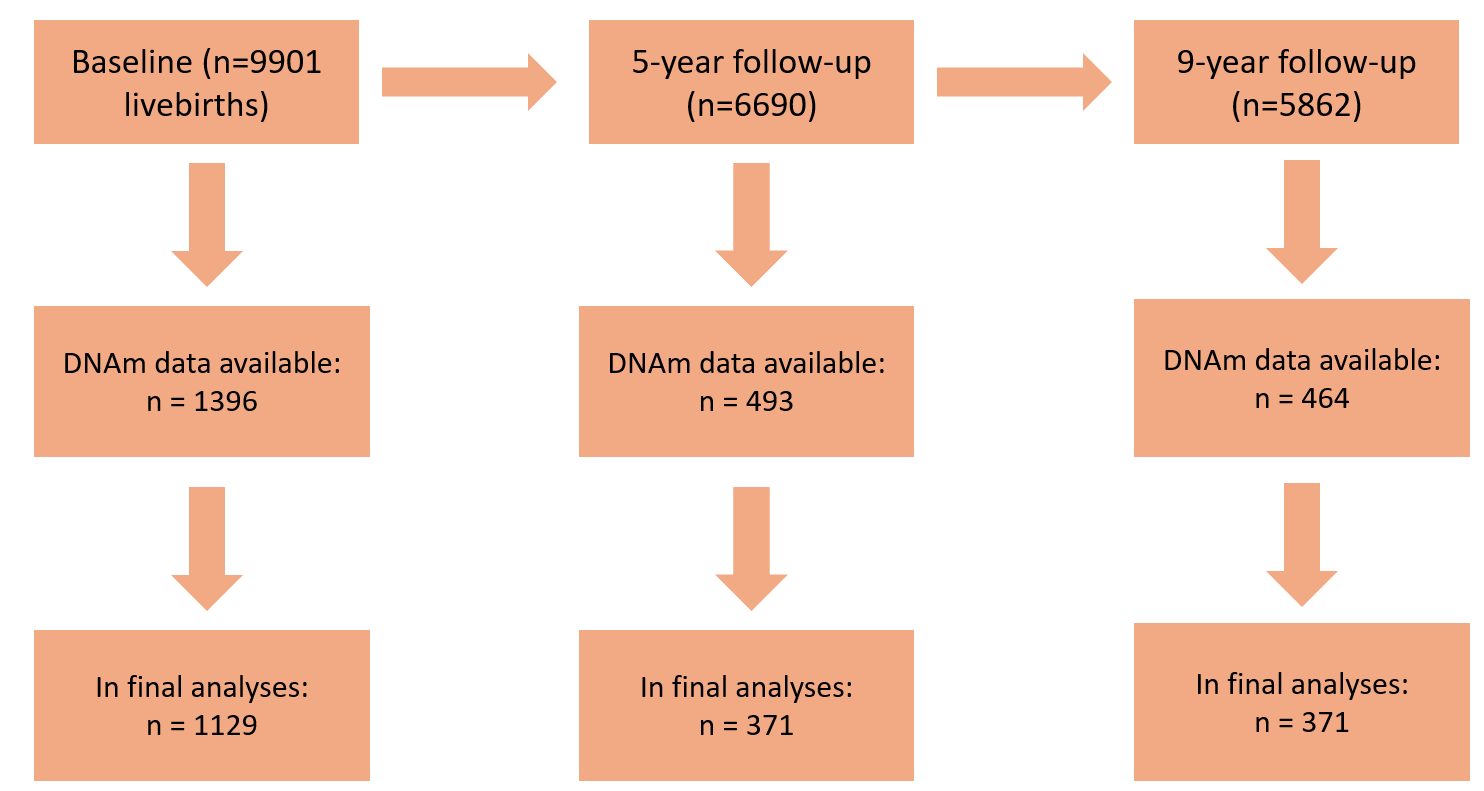


**References:**

1. Kooijman MN, Kruithof CJ, van Duijn CM, Duijts L, Franco OH, van IMH, et al. The Generation R Study: design and cohort update 2017. Eur J Epidemiol. 2016;31(12):1243-64
2. European Environmental Noise Directive. Directive 2002/49/EC (2002) <https://eur-lex.europa.eu/legal-content/EN/TXT/PDF/?uri=CELEX:32002L0049&from=en>
3. Schreurs EM, Jabben J, Verheijen ENG. Standard Model Instrumentation for Noise Assessments - STAMINA Model description. National Institute for Public Health and the Environment (2010)
4. Lehne B, Drong AW, Loh M, Zhang W, Scott WR, Tan ST, et al. A coherent approach for analysis of the Illumina HumanMethylation450 BeadChip improves data quality and performance in epigenome-wide association studies. Genome Biol. 2015;16(1):37
5. Reinius LE, Acevedo N, Joerink M, Pershagen G, Dahlen SE, Greco D, et al. Differential DNA methylation in purified human blood cells: implications for cell lineage and studies on disease susceptibility. PLoS One. 2012;7(7):e41361.
6. Gervin K, Salas LA, Bakulski KM, van Zelm MC, Koestler DC, Wiencke JK, et al. Systematic evaluation and validation of reference and library selection methods for deconvolution of cord blood DNA methylation data. Clin Epigenetics. 2019;11(1):125.

### HELIX

**Design and Study population**

Human Early Life Exposome (HELIX) study represents a collaborative project across six established and ongoing longitudinal population-based birth cohort studies in Europe: the Born in Bradford (BiB) study in the UK, the Étude des Déterminants pré et postnatals du développement et de la santé de l’Enfant (EDEN) study in France, the INfancia y Medio Ambiente (INMA) cohort in Spain, the Kaunus cohort (KANC) in Lithuania, the Norwegian Mother and Child Cohort Study (MoBa) and the RHEA Mother Child Cohort study in Crete, Greece. The HELIX project aims to measure and describe multiple environmental exposures from the different exposome domains during early life (pregnancy and childhood) and associate these with omics markers and child health outcomes (Maitre et al. 2018). For this study we used data from all cohorts except BiB (n=762), due to missing data for the exposure. For noise exposure during infancy we only had data available for MOBA and INMA (n=385). We used data from European ancestry children only. Models were adjusted for HELIX subcohort (EDEN, INMA, KANC, MoBa and RHEA).

*Ethical approval:* The six HELIX cohorts have the required permissions by national ethics committees for their cohort recruitment and follow-up visits and for secondary use of pre-existing samples and data. The work in HELIX was covered by new ethics approvals in each country. At enrolment in the HELIX project, families were asked to sign an informed consent form for the specific HELIX work including clinical examination and biospecimen collection and analysis. An Ethics Task Force was established to support the HELIX project on ethical issues, for advice on the project’s ethical compliance, identification and alerting to changes in legislation where applicable. Specific procedures are in place within HELIX to safeguard the privacy of study subjects and confidentiality of data.

**Road traffic noise exposure assessment**

We estimated road traffic noise levels at home addresses for the period from birth to visit at 6-11 years old (when DNA methylation was assessed), and for one year before visit at 6-11 years old. Road traffic noise levels were estimated using Lden, which is the annual average sound pressure level of a 24h period: day, evening, and night, with a 5 decibel (dB) penalty for evening noise (19:00-23:00, and 10 dB penalty added to nighttime noise (23:00-07:00). Lden was derived from noise maps produced in each local municipality under the European Noise Directive (EC Directive 2002/49/EC (EUR-Lex, n.d.)).

**Biological sample collection, DNA methylation quantification, quality control and normalization**

DNA, collected from buffy coat in EDTA tubes at age 7-9y, was extracted using the Chemagen kit (Perkin Elmer) in batches of 12 samples by the individual cohorts. DNA concentration was determined in a NanoDrop 1000 UV-Vis Spectrophotometer (ThermoScientific) and with Quant-iT™ PicoGreen® dsDNA Assay Kit (Life Technologies). DNA methylation was assessed with the Infinium HumanMethylatio450 beadchip (Illumina, USA) following manufacturer’s protocol at the University of Santiago de Compostela – Spanish National Genotyping Center (CeGen-USC, Spain). Bisulfite conversion of DNA carried out using the EZ 96-DNA kit (Zymo Research, USA) following the manufacturer’s standard protocol. All samples in the study were randomized taking into account sex, cohort and panel (samples from the same child collected at different time points). In addition, each plate contained a HapMap control sample and a total of 24 HELIX inter-plate duplicates were included.

DNA methylation data were pre-processed using the minfi package. Following guidelines of Lehne work (Lehne et al. 2015), we increased the stringency of the detection p-value threshold to 10E-16 and probes not reaching a 98% call rate were excluded. Two samples were filtered due to overall quality: one had a call rate <98% and the other did not pass QC parameters of the MethylAid package (van Iterson et al. 2014). Then, data was normalized with the functional normalization method, which also includes Noob background subtraction and dye-bias correction (Triche et al. 2013). After that, several quality control checks were performed. First, we checked sex consistency using the shinyMethyl package (Fortin et al. 2014) and two samples were excluded. Genetic consistency of duplicates and samples from the same participant was checked with the 450k genotypes. In addition, genetic consistency was evaluated in those samples that had GWAS data and two of them were excluded. Centered-correlation was around 0 for unrelated samples and around 0.8 for duplicates and panel samples. Principal component analysis showed no differential clusters, however a degree of grouping within the cluster was observed for some biological variables (sex, cohort) and for some technical variables. Duplicated samples and HapMap samples were removed as well as control probes, probes designed to detect SNPs and probes to measures methylation levels at non-CpG sites. The dataset used of the analysis was already corrected for the slide batch effect using the ComBat method (Leek et al. 2012). While applying ComBat main covariates were protected.

Proportion of six cord white blood cell types (CD4+ T-lymphocytes, CD8+ T-lymphocytes, NK (natural killer) cells, B-lymphocytes, monocytes and granulocytes) were computed using the Houseman method and the Reinius reference panel (Reinius et al. 2012)(Houseman et al. 2012).

**Acknowledgements and Funding**

The authors would like to thank all the participating children, parents, practitioners and researchers in the six countries who took part in this study.

The research leading to these results has received funding from the European Community’s Seventh Framework Programme (FP7/2007-206) under grant agreement no 308333—the HELIX project. The Norwegian Mother and Child Cohort Study (MoBa) is supported by the Norwegian Ministry of Health and the Ministry of Education and Research, NIH/NIEHS (contract no. N01-ES-75558), and NIH/NINDS (grant no. 1 UO1 NS 047537-01 and grant no. 2 UO1 NS 047537-06A1). The Rhea project was financially supported by European projects, and the Greek Ministry of Health (Program of Prevention of Obesity and Neurodevelopmental Disorders in Preschool Children, in Heraklion district, Crete, Greece: 2011–2014; 'Rhea Plus': Primary Prevention Program of Environmental Risk Factors for Reproductive Health, and Child Health: 2012–2015). We acknowledge support from the grant CEX2023-0001290-S funded by MCIN/AEI/ 10.13039/501100011033, and support from the Generalitat de Catalunya through the CERCA Program.


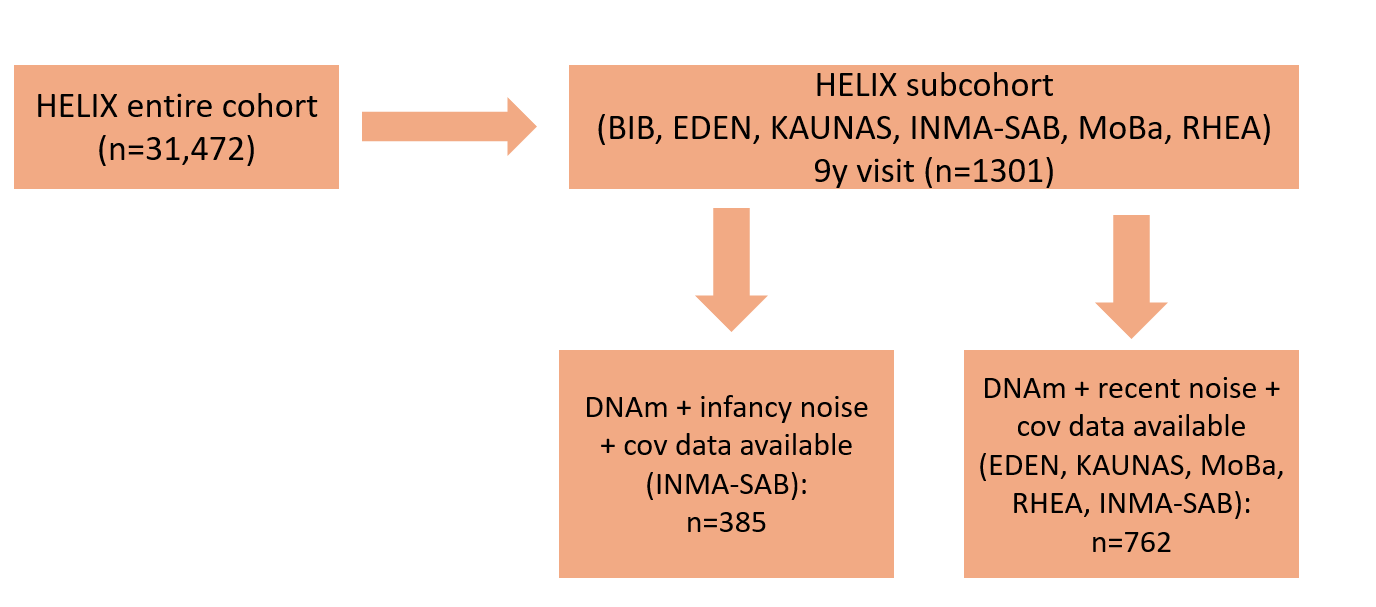


### INMA

**Design and Study population**

INfancia y Medio Ambiente (Environment and Childhood) study (INMA, <http://www.proyectoinma.org/>) is a collection of birth cohorts in Spain whose aim is to study the effect of pre- and postnatal environmental exposures (air pollutants, water, and diet during pregnancy) on growth, health, and development starting from early foetal life to adolescence (Guxens et al. 2012). The study enrolled pregnant women (12 weeks of pregnancy) at public primary health care centres or public hospitals between 1997 and 2008. Data on mothers and their children were collected through questionnaires, face-to-face interviews, clinical data, physical examination, and ultrasound measurements. Biological samples were also collected. The present study used data from participants of European ancestry recruited between 2004 and 2006 in the de novo cohort sited in Sabadell (n=348 at birth, n=177 and 162 at 4y for the infancy and recent noise exposures).

The study was approved by the Ethics Committee of the reference hospital, and all participants gave their written informed consent.

**Road traffic noise exposure assessment**

Available noise map developed in 2006 for Sabadell were used to estimate the annual average levels of noise at each participant’s geocoded residential address. We selected the map of 2006 because it corresponded to the study periods of interest. The map complied with the requirements of the European Environmental Noise Directive (European Environmental Noise [Directive, 2002](https://www.sciencedirect.com/science/article/pii/S0160412023006876?via%3Dihub#b0050)). Noise was measured using a street categorization method considering the different types of street and land uses. Additionally, street geometry, presence of activities, type of traffic, and traffic flow were included to determine the noise level (European Environmental Noise [Directive, 2002](https://www.sciencedirect.com/science/article/pii/S0160412023006876?via%3Dihub#b0050)). The map was constructed to estimate the residential noise levels at the most exposed façade at a height of 4 meters.

To estimate road traffic noise exposure, we used the day-evening-night EU noise indicator (L_DEN_). L_DEN_ represents the A-weighted average sound level over the entire 24-hour day with penalties for the evening (+5dB) and the night (+10dB), as suggested by the Environmental Noise Directive to take into consideration the expected greater health impact of the evening and night-time periods (European Environmental Noise Directive, 2002). The levels of LDEN were calculated at each geocoded address that the participants had resided at during the periods of interest. We assigned the noise of the street closest to the geocode at a maximum distance of 50 meters. Average noise levels were calculated for each participant for each period of interest, taking into account the number of days that a participant spent at each address when more than one address was available.

**Biological sample collection, DNA methylation quantification, quality control and normalization**

Cord blood and whole blood collected at age 4y were extracted using the Chemagen kit (Perkin Elmer). DNA concentration was determined by a NanoDrop spectrophotometer (Thermo Scientific) and with the Quant-iT PicoGreen dsDNA Assay Kit (Life Technologies).

Blood methylation data was produced in two laboratories: the Genome Analysis Facility of the University Medical Center Groningen (UMCG) in Holland as part of the MeDALL project (0y and 4y), and the Bellvitge Biomedical Research Institute (IDIBELL) in Barcelona as part of the BREATHE project (0y). Both laboratories randomized the samples in batches and followed the Illumina protocol for the Infinium HumanMethylation450 BeadChip. Briefly, 500 ng of DNA was bisulfite-converted using the EZ 96-DNA methylation kit, and DNA methylation was measured through hybridization on the BeadChips. BeadChips were scanned with an Illumina iScan, and image data were uploaded into the Methylation Module of Illumina’s analysis software GenomeStudio and converted in β-values.

Two blood samples with overall low quality (MethylAid package) (van Iterson et al. 2014) and three blood samples discordant for sex (shinyMethyl package) (Fortin et al. 2014) were removed. After applying a stringent detection p-value of 1.10E-16 (Lehne et al. 2015), 18 blood samples with a call rate <98% were excluded. We removed 7,136 probes with a call rate <95%, control probes, and SNP probes designed to detect genetic polymorphisms. Data was normalized with the functional normalization method with prior background correction with Noob implemented in the minfi package (Aryee et al. 2014). All the samples were quality controlled together, regardless of the laboratory of processing, and the laboratory batch effect was eliminated with the ComBat method (Leek et al. 2012). While applying ComBat main covariates were protected.

For cord blood, proportions of seven cord blood cell types (CD4+ T-lymphocytes, CD8+ T-lymphocytes, NK (natural killer) cells, B-lymphocytes, monocytes, granulocytes and nucleated red blood cells (nRBC)) were computed by applying the Gervin reference panel (Gervin et al. 2019). For child blood, proportions of six blood cell types (CD4+ T-lymphocytes, CD8+ T-lymphocytes, NK (natural killer) cells, B-lymphocytes, monocytes, granulocytes) were estimated with the Houseman method using Reinius’ reference (Reinius et al. 2012)(Houseman et al. 2012).

**Acknowledgements and Funding**

This study was funded by grants from Instituto de Salud Carlos III (Red INMA G03/176; CB06/02/0041; PI041436; PI17/01340 incl. FEDER funds, PI081151 incl. FEDER funds), Generalitat de Catalunya-CIRIT 1999SGR 00241, Fundació La marató de TV3 (090430), EU Commission (261357-MeDALL: Mechanisms of the Development of ALLergy), and European Research Council (268479-BREATHE: BRain dEvelopment and Air polluTion ultrafine particles in scHool childrEn). This publication was co-financed by the Agencia Estatal de Investigación (AEI) and the European Social Fund (FSE) " EL FSE invierte en tu futuro" with reference number PRE2020-092005, according to the Resolution of the Presidency of the AEI, by which grants are awarded for pre-doctoral contracts for the training of doctors, call 2020 (awarded to M.S.W.K). We also acknowledge support from the grant CEX2023-0001290-S funded by MCIN/AEI/ 10.13039/501100011033, and support from the Generalitat de Catalunya through the CERCA Program, and from the Ministry of Research and Universities of the Government of Catalonia (2021 SGR 01564).


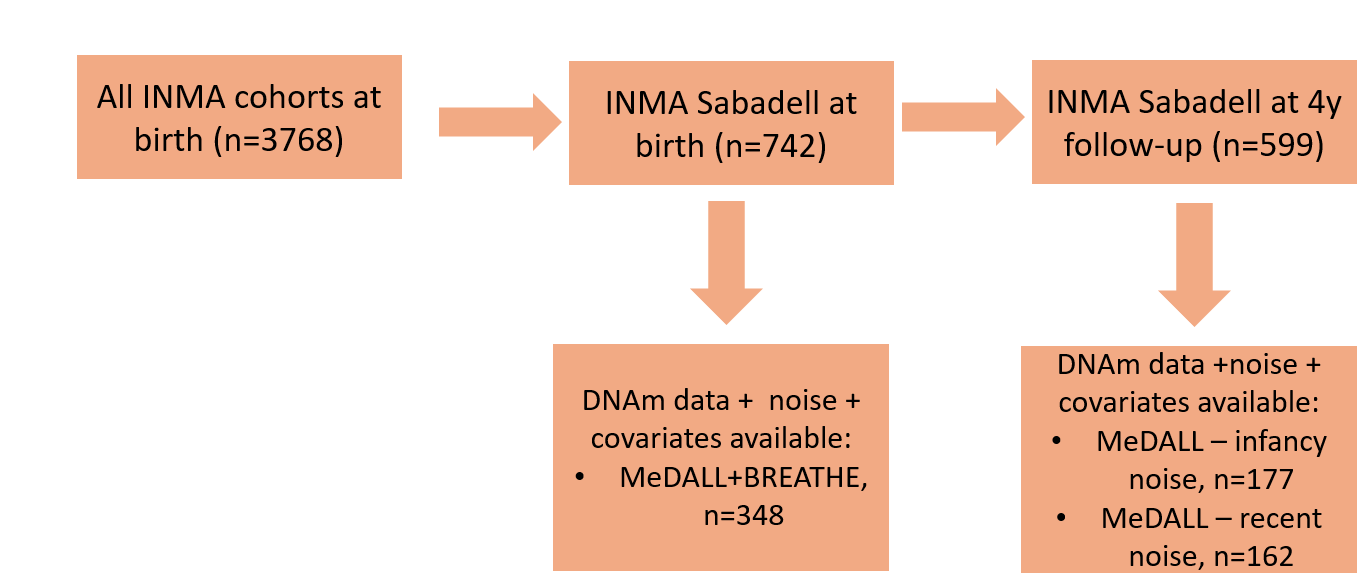


**References**

Aryee MJ, Jaffe AE, Corrada-Bravo H, Ladd-Acosta C, Feinberg AP, Hansen KD, et al. 2014. Minfi: A flexible and comprehensive Bioconductor package for the analysis of Infinium DNA methylation microarrays. Bioinformatics; doi:10.1093/bioinformatics/btu049.

Fortin J-P, Fertig E, Hansen K. 2014. shinyMethyl: interactive quality control of Illumina 450k DNA methylation arrays in R. F1000Research 3:175; doi:10.12688/f1000research.4680.2.

Gervin K, Salas LA, Bakulski KM, Van Zelm MC, Koestler DC, Wiencke JK, et al. 2019. Systematic evaluation and validation of reference and library selection methods for deconvolution of cord blood DNA methylation data. Clinical Epigenetics 11:1–15; doi:10.1186/s13148-019-0717-y.

Guxens M, Ballester F, Espada M, Fernandez MF, Grimalt JO, Ibarluzea J, et al. 2012. Cohort Profile: the INMA--INfancia y Medio Ambiente--(Environment and Childhood) Project. Int J Epidemiol 41: 930–940.

Houseman E, Accomando WP, Koestler DC, Christensen BC, Marsit CJ, Nelson HH, et al. 2012. DNA methylation arrays as surrogate measures of cell mixture distribution. BMC Bioinformatics 13:86; doi:10.1186/1471-2105-13-86.

Leek JT, Johnson WE, Parker HS, Jaffe AE, Storey JD. 2012. The SVA package for removing batch effects and other unwanted variation in high-throughput experiments. Bioinformatics; doi:10.1093/bioinformatics/bts034.

Lehne B, Drong AW, Loh M, Zhang W, Scott WR, Tan S-T, et al. 2015. A coherent approach for analysis of the Illumina HumanMethylation450 BeadChip improves data quality and performance in epigenome-wide association studies. Genome Biology 16:37; doi:10.1186/s13059-015-0600-x.

Maitre L, De Bont J, Casas M, Robinson O, Aasvang GM, Agier L, et al. 2018. Human Early Life Exposome (HELIX) study: A European population-based exposome cohort. BMJ Open; doi:10.1136/bmjopen-2017-021311.

Reinius LE, Acevedo N, Joerink M, Pershagen G, Dahlén S-E, Greco D, et al. 2012. Differential DNA Methylation in Purified Human Blood Cells: Implications for Cell Lineage and Studies on Disease Susceptibility. A.H. Ting, ed PLoS ONE 7:e41361; doi:10.1371/journal.pone.0041361.

Triche TJ, Weisenberger DJ, Van Den Berg D, Laird PW, Siegmund KD. 2013. Low-level processing of Illumina Infinium DNA Methylation BeadArrays. Nucleic Acids Research 41; doi:10.1093/nar/gkt090.

van Iterson M, Tobi EW, Slieker RC, den Hollander W, Luijk R, Slagboom PE, et al. 2014. MethylAid: visual and interactive quality control of large Illumina 450k datasets. Bioinformatics (Oxford, England) 30:3435–7; doi:10.1093/bioinformatics/btu566.

### LISA

**Design and Study population**

The influence of Life-style factors on the development of the Immune System and Allergies in East and West Germany (LISA) study is a population-based birth cohort study. A total of 3,094 healthy, full-term neonates were recruited between 1997 and 1999 in Munich, Leipzig, Wesel and Bad Honnef. Detailed descriptions of the LISA study have been published elsewhere (1). Approval was given by the local Ethics Committees and written consent from participant’s families was obtained.

**Road traffic noise exposure assessment**

Road traffic noise data is based on the Munich noise map, which was created for the year 2007. Details on the road traffic noise modelling procedure can be found elsewhere (2). CadnaA software (“Computer Aided Noise Abatement”) was used for the calculations based on a 3-dimensional terrain model to account for multiple reflections and shielding from objects, including houses and other noise barriers. Modifying effects of traffic noise protection measures such as noise shield walls and also of buildings were considered in the noise model. Noise indicators defined according to the European Environmental Noise Directive and its implementation into German law-the 34th Federal Immission Control Ordinance (34. BImSchV, 2006) (3) were available. For this project, the day–evening–night noise indicator *L*_den_ was used to assess overall noise annoyance accounting for increased levels of disturbance by noise during the evening and night times. More details are provided elsewhere (4).

**Biological sample collection, DNA methylation quantification, quality control and normalization**

Samples using genomic DNA (gDNA) from blood-clots were collected at the age of 6 and 10 years. DNAm was measured using the MethylationEPIC BeadChip (Illumina, Inc., San Diego, CA). We excluded samples with a bad-sample cut-off under 10.0 of the log median intensities of both methylated and unmethylated channels as suggested in the minfi package (5). Functional normalization with five principal components based on Fortin et al. (6) was used to normalize the data. Outliers were removed if they were detected as critical by at least two of the following detection methods: detectOutlier() in the lumi package (7), pcout() or pfilter from the wateRmelon package (8), locfdr as reported by Hannum et al. (9) or based on outliers in the first 3 PCs. Bad probes were removed, if they had missing values or the detection p-value was above 0.01 in more than 1% of the samples. Technical correction was done using ComBat from Johnson et al. (10) for Plate, Chip and Position. Cell type proportions were estimated with the Houseman method (11). More details can be found elsewhere (12).

**Acknowledgements and Funding**

The LISA study was mainly supported by grants from the Federal Ministry for Education, Science, Research and Technology and in addition from Helmholtz Zentrum Munich (former GSF), Helmholtz Centre for Environmental Research - UFZ, Leipzig, Research Institute at Marien-Hospital Wesel, Pediatric Practice, Bad Honnef for the first 2 years. The 4 year, 6 year, 10 year and 15 year follow-up examinations of the LISA study were covered from the respective budgets of the involved partners (Helmholtz Zentrum Munich (former GSF), Helmholtz Centre for Environmental Research - UFZ, Leipzig, Research Institute at Marien-Hospital Wesel, Pediatric Practice, Bad Honnef, IUF – Leibniz-Research Institute for Environmental Medicine at the University of Düsseldorf) and in addition by a grant from the Federal Ministry for Environment (IUF Düsseldorf, FKZ 20462296). Further, the 15-year follow-up examination of the LISA study was supported by the Commission of the European Communities, the 7th Framework Program: MeDALL project.

The authors thank all the families for their participation in the LISA study. Furthermore, we thank all members of the LISA Study Group for their excellent work. The LISA Study group consists of the following: Institute of Epidemiology, Helmholtz Zentrum München, German Research Center for Environmental Health, Neuherberg (Standl M, Heinrich J, Schulz H, Ferland M, Flexeder C, Zeller C, Thiering E, Harris C); Department of Pediatrics, Marien-Hospital, Wesel (von Berg A, Berdel D, Gappa M); Pediatric Practice, Bad Honnef (Schaaf B); Helmholtz Centre of Environmental Research – UFZ, Department of Environmental Immunology/Core Facility Studies, Leipzig (Herberth G, Bauer M, Röder S, Schilde M). We further want to thank Nadine Lindemann for her work analyzing the DNA methylation samples.


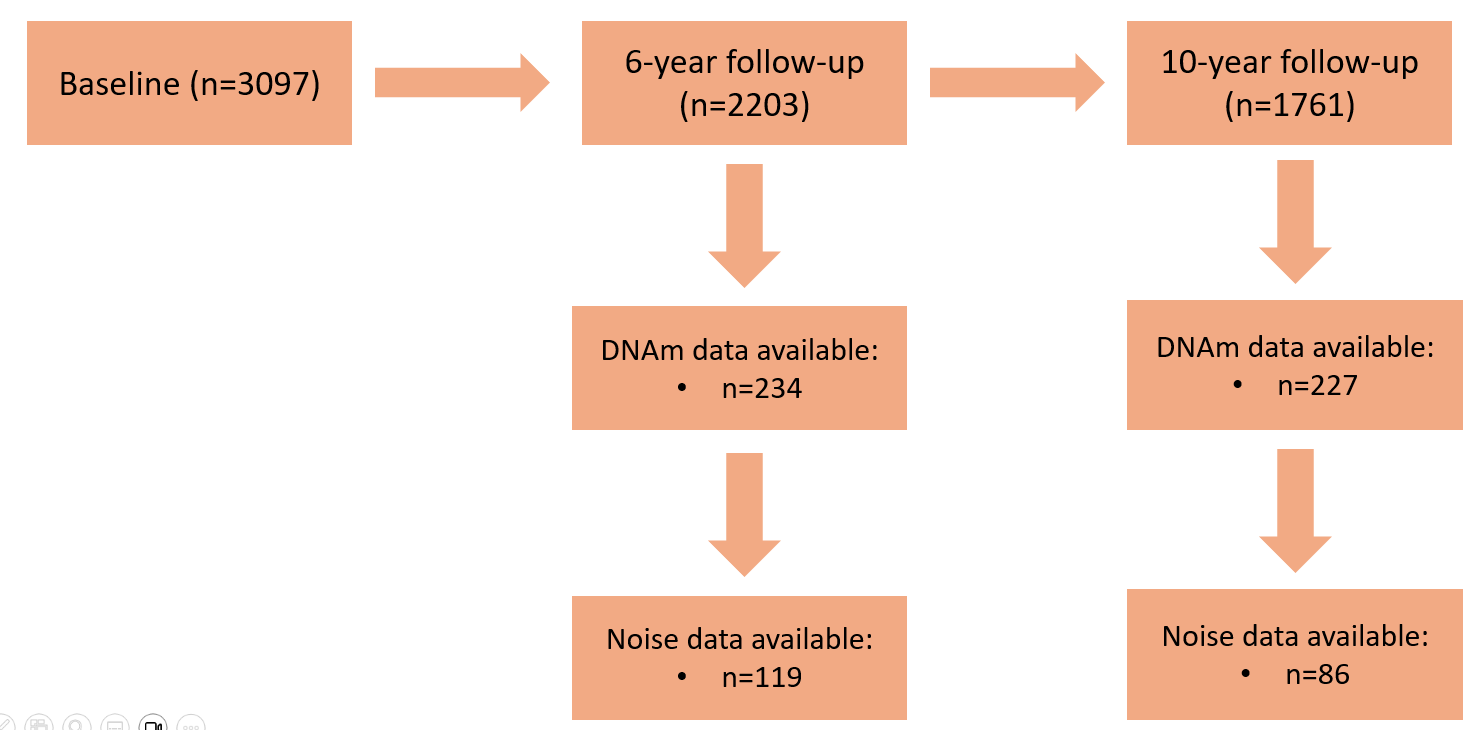


**Reference**

1. Heinrich J, Bolte G, Hölscher B, Douwes J, Lehmann I, Fahlbusch B, et al. Allergens and endotoxin on mothers' mattresses and total immunoglobulin E in cord blood of neonates. Eur Respir J. 2002;20(3):617-23
2. Birk, M., et al., 2011. Road traffic noise: self-reported noise annoyance versus GIS modelled road traffic noise exposure. J. Environ. Monit. 13, 3237–3245.
3. 34. BImSchV. Vierunddreißigste Verordnung zur Durchführung des Bundes-Immissionsschutzgesetzes (Verordnung über die Lärmkartierung) vom 6. März, 2006. BGBl. I:516.
4. Tiesler CMT, Birk M, Thiering E, Kohlböck G, Koletzko S, Bauer C-P, et al. Exposure to road traffic noise and children's behavioural problems and sleep disturbance: results from the GINIplus and LISAplus studies. Environ Res 2013;123:1–8.
5. Minfi: a flexible and comprehensive Bioconductor package for the analysis of Infinium DNA methylation microarrays | Bioinformatics | Oxford Academic. Accessed September 2, 2021. https://academic.oup.com/bioinformatics/article/30/10/1363/267584
6. Fortin JP, Labbe A, Lemire M, et al. Functional normalization of 450k methylation array data improves replication in large cancer studies. Genome Biol. 2014;15(12):503. doi: 10.1186/s13059-014-0503-2
7. Du P, Kibbe WA, Lin SM. lumi: a pipeline for processing Illumina microarray. *Bioinforma Oxf Engl*. 2008;24(13):1547-1548. doi:10.1093/bioinformatics/btn224
8. Pidsley R, Y Wong CC, Volta M, Lunnon K, Mill J, Schalkwyk LC. A data-driven approach to preprocessing Illumina 450K methylation array data. *BMC Genomics*. 2013;14:293. doi:10.1186/1471-2164-14-293
9. Hannum G, Guinney J, Zhao L, et al. Genome-wide methylation profiles reveal quantitative views of human aging rates. *Mol Cell*. 2013;49(2):359-367. doi:10.1016/j.molcel.2012.10.016
10. Johnson WE, Li C, Rabinovic A. Adjusting batch effects in microarray expression data using empirical Bayes methods. Biostat Oxf Engl. 2007;8(1):118–127. doi: 10.1093/biostatistics/kxj037
11. Houseman, E.A.*, et al.* DNA methylation arrays as surrogate measures of cell mixture distribution. *BMC bioinformatics* **13**, 86 (2012)
12. Kilanowski A, Chen J, Everson T, Thiering E, Wilson R et al. Methylation risk scores for childhood aeroallergen sensitization: Results from the LISA birth cohort. Allergy 2022

### PIAMA

**Design and Study population**

For the PIAMA (Prevention and Incidence of Asthma and Mite Allergy) birth cohort study, pregnant women were recruited in 1996-1997 during their second trimester of pregnancy from a series of communities in the North, West, and Centre of The Netherlands. Details of the study design have been published previously [1,2]. Non-allergic pregnant women were invited to participate in a “natural history” study arm. Pregnant women identified as allergic through a validated screening questionnaire were primarily allocated to an intervention arm with a random subset allocated to the natural history arm. The intervention involved the use of mite-impermeable mattress and pillow covers. The study started with 3,963 newborns. Full questionnaire follow-ups of the children took place at 3 months of age, yearly from 1 to 8 years of age, and at ages 11 and 14 years.Medical examinations were performed in subsets of the population at ages 4, 8, 12 and 16 years. DNA was extracted of children who provided blood samples at ages 4 and 8 years. The Medical Ethical Committees of the participating institutes approved the study, and all participants gave written informed consent.

**Road traffic noise exposure assessment**

Annual average road traffic and railway noise exposure have been estimated using the STAMINA model (Standard Model Instrumentation for Noise Assessments), which has been developed at the Dutch National

Institute for Public Health and the Environment (3]. Daily average (L_den_) and nighttime average (L_night_) road traffic and railway noise exposure at the adolescents' home addresses were estimated for 2011. Lden is the A-weighted noise level over a whole day with a penalty of 5 dB(A) for evening noise (19.00–23.00) and a penalty of 10 dB(A) for nighttime noise (23.00–07.00). It does not capture occasional very high noise levels (noise peaks). Because L_den_ and Lnight were highly correlated (*r*=0.99 for road traffic noise; *r*=0.95 for railway noise), we only included Lden in this analysis.

**Biological sample collection, DNA methylation quantification, quality control and normalization**

DNA methylation measurement

DNA from peripheral blood samples was extracted using the QIAamp blood kit (Qiagen or equivalent protocols), followed by precipitation-based concentration using GlycoBlue (Ambion). DNA concentration was determined by Nanodrop measurement and Picogreen quantification. 500 ng of DNA was bisulphite-converted using the EZ 96-DNA methylation kit (Zymo Research), following the manufacturer’s standard protocol. After verification of the bisulphite conversion step using Sanger Sequencing, DNA concentration was normalized and the samples were randomized to avoid batch effects. All paired samples were hybridized on the same chip. Standard male and female DNA samples were included in this step for quality control.

DNA methylation was measured at the Genome Analysis Facility, Department of Genetics, University Medical Center Groningen, Groningen, the Netherlands as part of the MedALL project[4]

Quality control and pre-processing of 450k data

DNA methylation data were pre-processed in R with the Bioconductor package Minfi6, using the original IDAT files extracted from the HiScanSQ scanner. Samples that did not provide significant methylation signals in more than 10% of probes (detection P=0.01) were excluded from further analysis. Samples were also excluded in cases of low staining efficiency, low single base extension efficiency, low stripping efficiency of DNA from probes after single base extension, poor hybridization performance, poor bisulphite conversion and high negative control probe staining. Further, we used the 65 SNP probes to check for concordances between paired DNA samples from the sample individual and assessed the methylation distribution of the X-chromosome to verify gender. Paired samples with Pearson correlation coefficients <0.9 were regarded as sample mix-ups and were excluded from the study. In probe filtering, we excluded probes on sex chromosomes, probes that mapped on multi-loci, the 65 random SNPs assay and probes that contained SNPs at the target CpG sites with a minor allele frequency >10%. The allele frequencies of a list of SNPs were obtained from 1000 Genomes, release 20110521 for the CEU population. Finally, to correct Type I and Type II bias, we implemented “DASEN” to perform signal correction and normalization. (ref Xu et al, PMID: 29496485)

**Acknowledgements and Funding**

The PIAMA study was supported by The Netherlands Organization for Health Research and Development; The Netherlands Organization for Scientific Research; Lung Foundation Netherlands (formerly The Netherlands Asthma Fund); The Netherlands Ministry of Spatial Planning, Housing, and the Environment; The Netherlands Ministry of Health, Welfare, and Sport; and the National Institute of Public Health and the Environment (RIVM). DNA methylation analysis was supported by the European Union (FP7, MeDAll study).


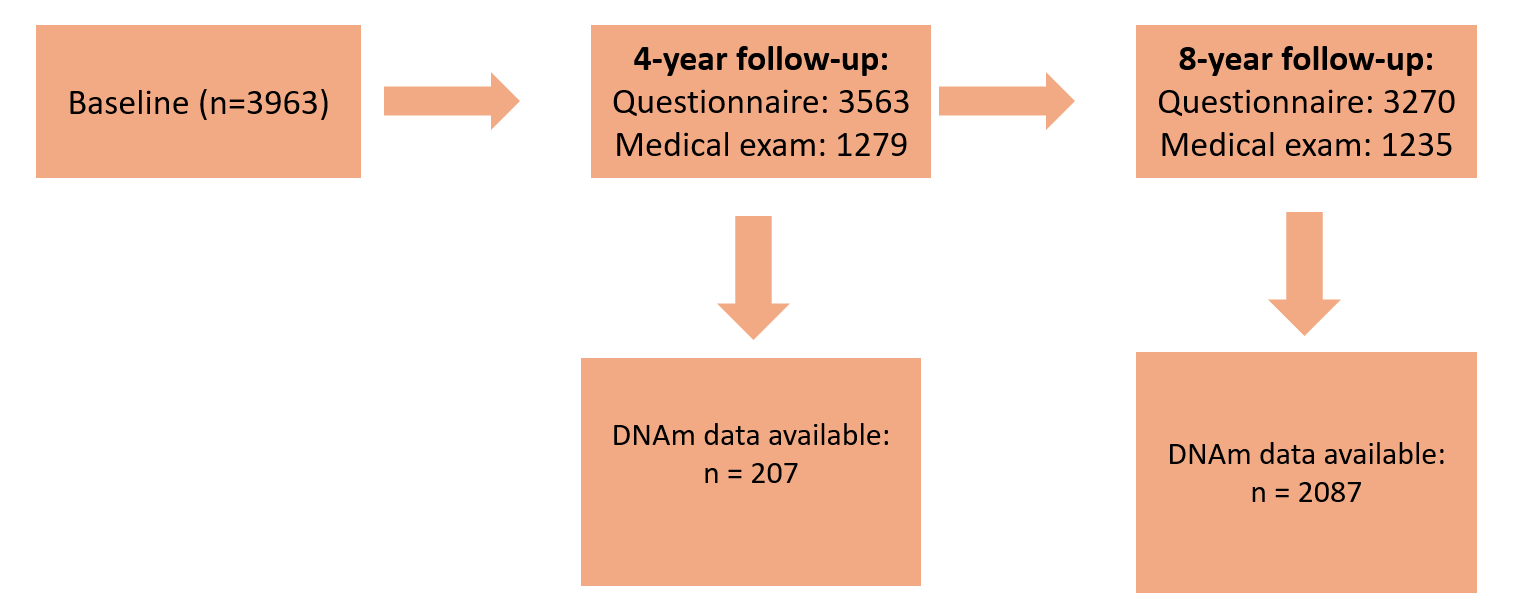


**References**

1. Brunekreef B, Smit J, de Jongste J, Neijens H, Gerritsen J, Postma D, Aalberse R, Koopman L, Kerkhof M, Wijga A, van Strien R. The prevention and incidence of asthma and mite allergy (PIAMA) birth cohort study: design and first results. Pediatr Allergy Immunol 2002: 13 Suppl 15: 55-60.

2. Wijga AH, Kerkhof M, Gehring U, de Jongste JC, Postma DS, Aalberse RC, Wolse AP, Koppelman GH, van Rossem L, Oldenwening M, Brunekreef B, Smit HA. Cohort profile: the prevention and incidence of asthma and mite allergy (PIAMA) birth cohort. Int J Epidemiol 2014: 43(2): 527-535.

3. Schreurs, E.M., Jabben, J., Verheijen, E.N.G., 2010. STAMINA - model description. Standard model instrumentation for noise. Retrieved from Bilthoven. https://www.rivm.nl/en/Documents_and_publications/Scientific/Reports/2010/maart/STAMINA_Model_description_Standard_Model_Instrumentation_for_Noise_Assessments.

4. Xu C-J, Söderhäll C, Bustamante M, *et al.* DNA methylation in childhood asthma: an epigenome-wide meta-analysis. *Lancet Respir Med* 2018; **6**: 379–88.

### Differentially methylated region (DMR) analysis input parameters

Input parameters used in the comb-p algorithm:

Parameter Value Description

dist 1000 Maximum distance to search for adjacent peaks.

seed 0.05 A value must be at least this large/small in order to seed a region.

region-filter-p 0.05 Maximum adjusted region-level p-value to be reported in final output.

region-filter-n 2 Require at least this many probes for a region to be reported in final output.

Input parameters used in the DMRcate algorithm:

Parameter Value Description

lambda 1000 Gaussian kernel bandwidth for smoothed-function estimation.

Gaps ≥ lambda between significant CpG sites will be in separate DMRs.

C 2 Scaling factor for bandwidth. Gaussian kernel is calculated

where lambda/C = sigma. Empirical testing shows that, for 450k data when lambda = 1000, near-optimal prediction of sequencing-derived DMRs is obtained when C is approximately 2.

Pcutoff 0.05 p-value cutoff to determine DMRs.

min.cpgs 2 Minimum number of consecutive CpGs constituting a DMR.

### Table 1 Number of probes removed during the quality control for the meta-analysis

| Time window | Prenatal | Infancy&Children 4-6 | Infancy&Children 8-10 | Recent&Children 4-6 | Recent&Children 8-10 |
| --- | --- | --- | --- | --- | --- |
| Initial number of probes | 479643 | 483863 | 484117 | 483391 | 484466 |
| Cross-reactive probes | 38274 | 38539 | 38709 | 38434 | 38741 |
| Probes on sex chromosomes | 9881 | 9881 | 10225 | 9881 | 10225 |
| Probes only existed in one cohort | 11810 | 650 | 1398 | 1380 | 635 |
| Final number of probes | 419669 | 434793 | 434785 | 433696 | 434865 |

Table 2 Sample size of each cohort contributing to the meta-analysis

| Cohort | Cord blood | Children age 4-6 | | Children age 8-10 | |
| --- | --- | --- | --- | --- | --- |
|  | Prenatal period | Infancy | Recent | Infancy | Recent |
| BAMSE (epigene) | NA | NA | NA | 375 | 375 |
| BAMSE (MeDALL) | NA | 255 | 253 | 264 | 264 |
| GenR | 1129 | 371 | 265 | 371 | 371 |
| HELIX | NA | NA | NA | 385 | 762 |
| INMA | 348 | 177 | 162 | NA | NA |
| LISA | NA | 119 | NA | NA | 86 |
| PIAMA | NA | 207 | 207 | 207 | 207 |
| **Total** | 1477 | 1129 | 887 | 1602 | 2065 |


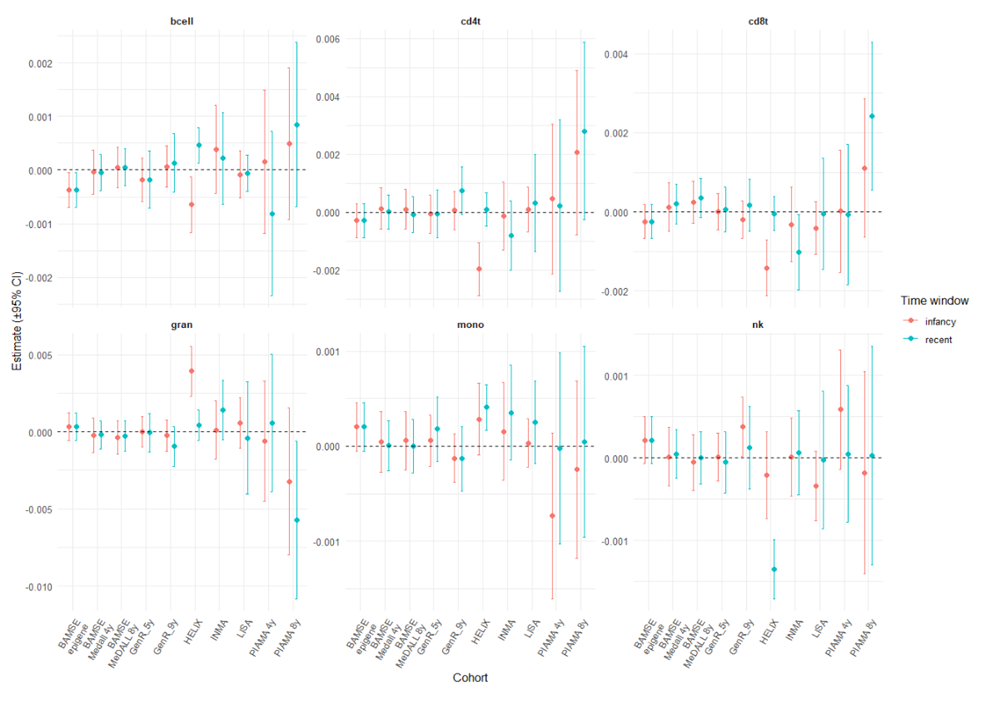


### Associations between road traffic noise exposure and cell types estimated using the Houseman method


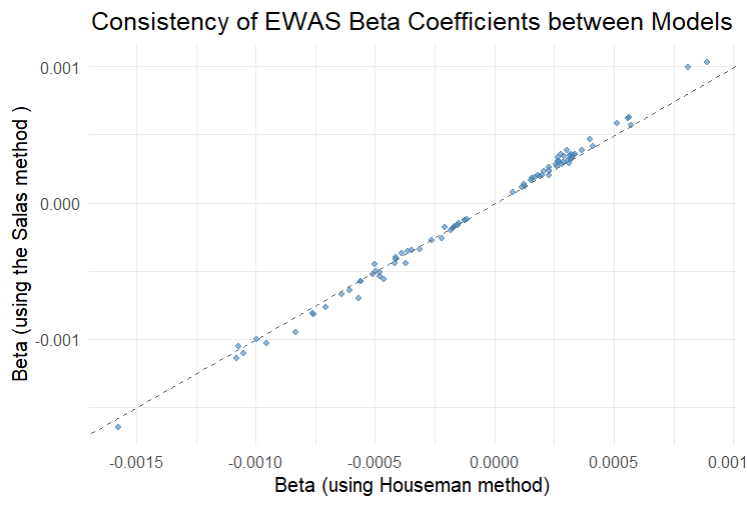


Comparison for the beta coefficients derived from the EWAS models adjusting for 6 cell types (Houseman) and 12 cell types (Salas). Model was conducted in the BAMSE Epigene dataset using the recent noise as exposure, only the CpGs with p value<10-4 were presented in the figure. Dash line indicating the line y=x.
