# Supplementary material for: Road traffic noise exposure and blood DNA methylation at birth and in childhood: An epigenome-wide meta-analysis
Source: Environ Int. Author manuscript; Available in PMC 2026 Jan 23. (PMC12829588; doi:10.1016/j.envint.2025.109976)
Supplement: Supplementary figures [file NIHMS2138169-supplement-Supplementary_figures.docx]

**Road traffic noise exposure and blood DNA methylation at birth and in childhood: An epigenome-wide meta-analysis**

**Supplemental Figures**

**Supplemental Figure 1** QQ plot in the discovery EWAS meta-analysis.

**Supplemental Figure 2** Meta-analysis and cohort-specific estimates for the suggestive DMPs.

## **Supplemental Figure 3** Comparisons of the effect estimates for the suggestive DMPs in the main model and sensitivity analysis additionally adjusted for maternal smoking during pregnancy

**Supplemental Figure 4** Leave-one-out meta-analysis for the suggestive DMPs in the discovery meta-analysis.

## **Supplemental Figure 5** Estimates for the suggestive DMP in the BAMSE Epigene, PIAMA, Generation R, LISA additionally adjusted for PM2.5 and black carbon

**Supplemental Figure 6** Cross look up of the suggestive DMPs in all time windows

## **Supplemental Figure 7** Tissue- or cell-specific signals for the suggestive significant DMPs from the eFORGE database

## **Supplemental Figure 8** Tissue- or cell-specific signals for all the CpGs within the significant DMRs from the eFORGE database


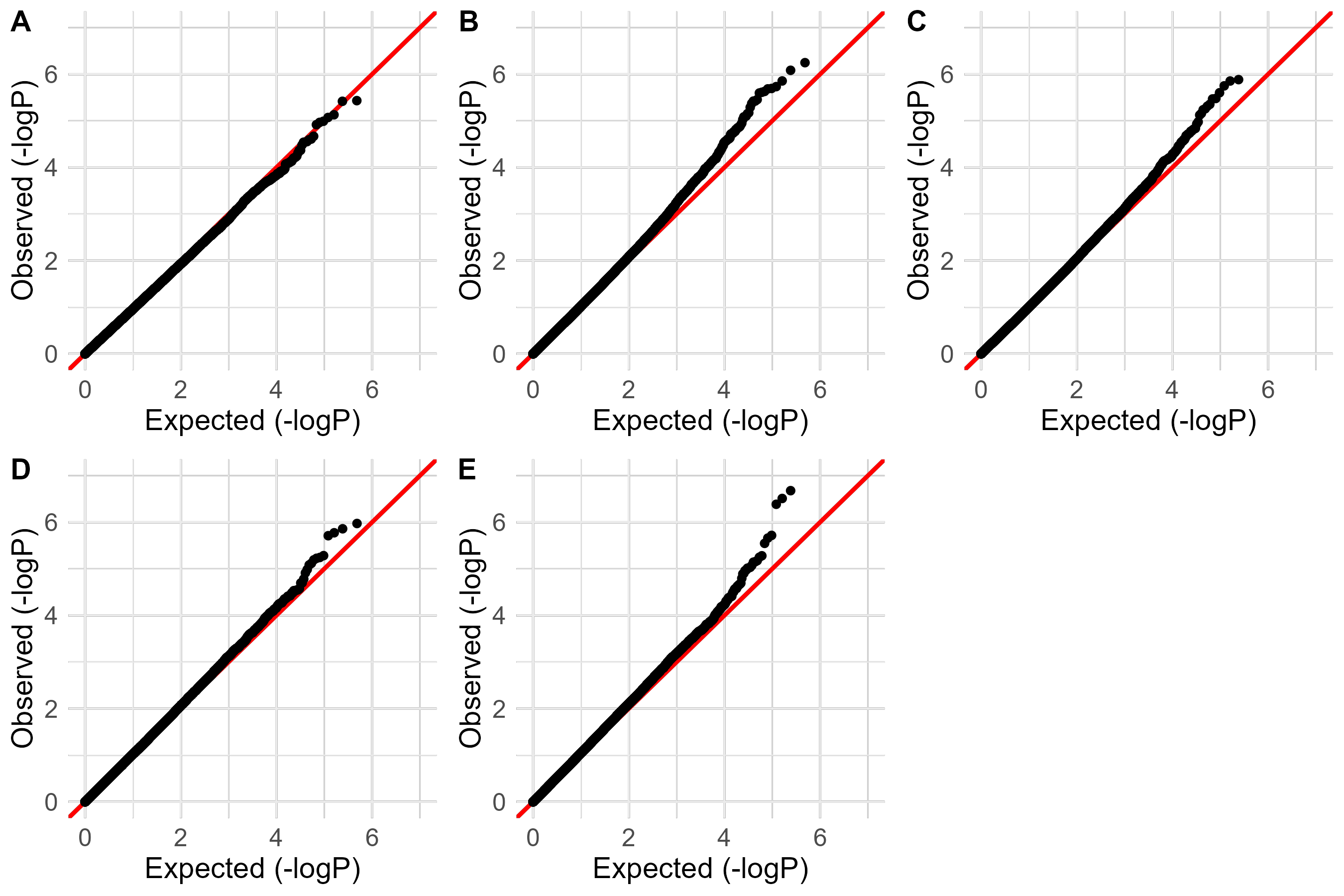


Supplemental Figure 1 QQ plot in the discovery EWAS meta-analysis. (A) Prenatal noise exposure and DNA methylation in cord blood (B) Infancy noise exposure and DNA methylation in children’s blood at 4-6 years; (C) Recent noise exposure and DNA methylation in children’s blood at 4-6 years; (D) Infancy noise exposure and DNA methylation in children’s blood at 8-10 years; (E) Recent noise exposure and DNA methylation in children’s blood at 8-10 years.

**A**


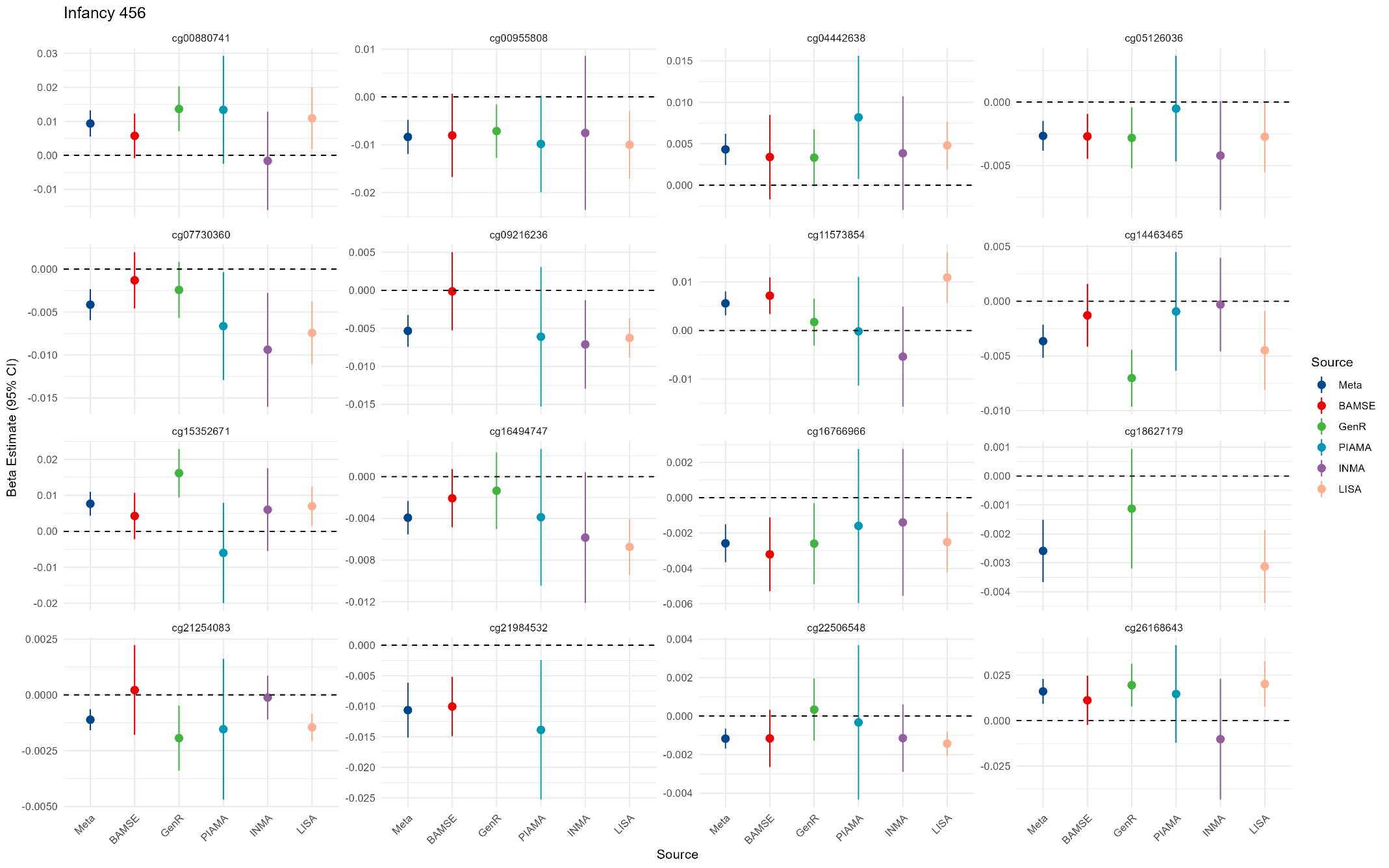


**B**


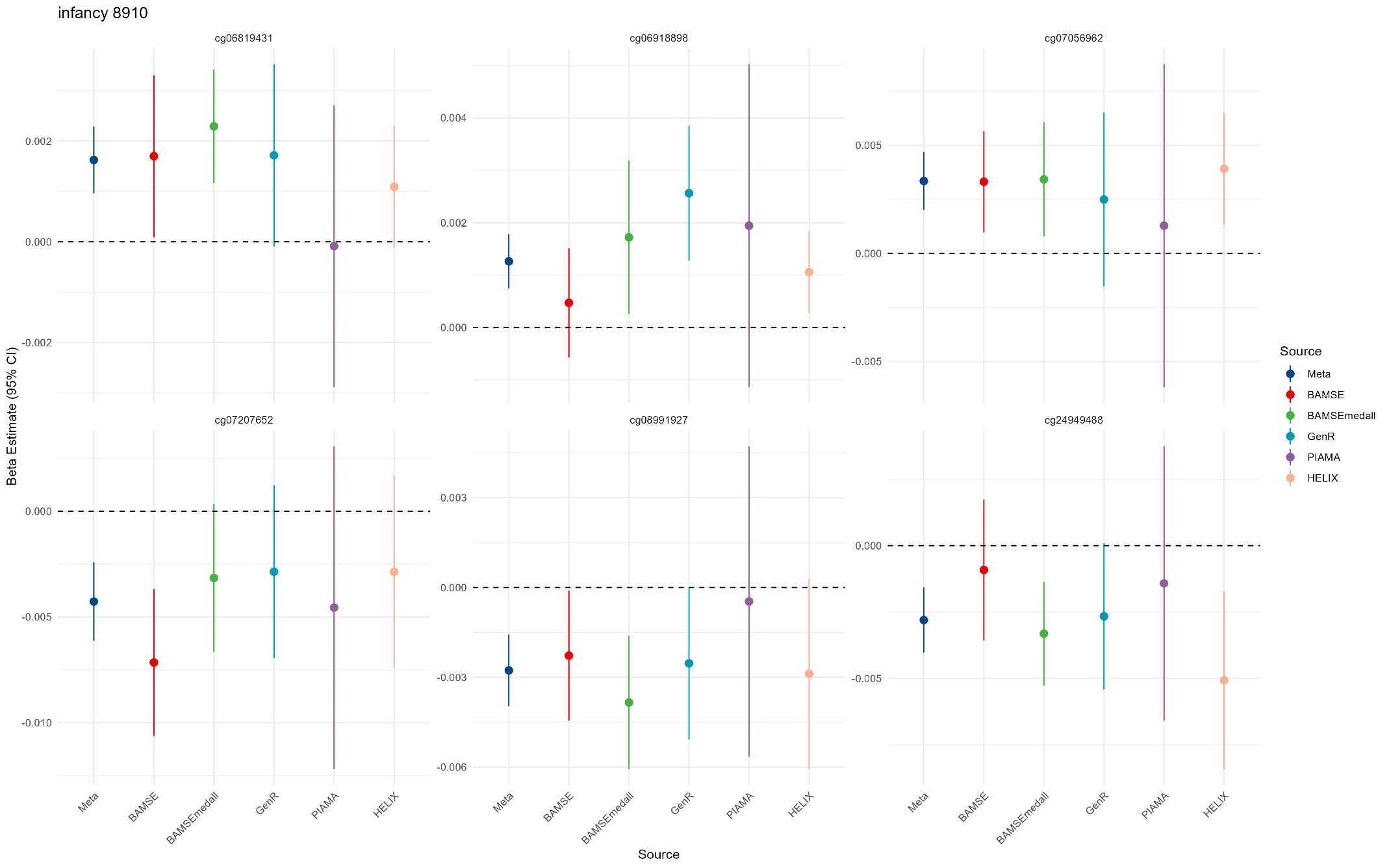


**C**


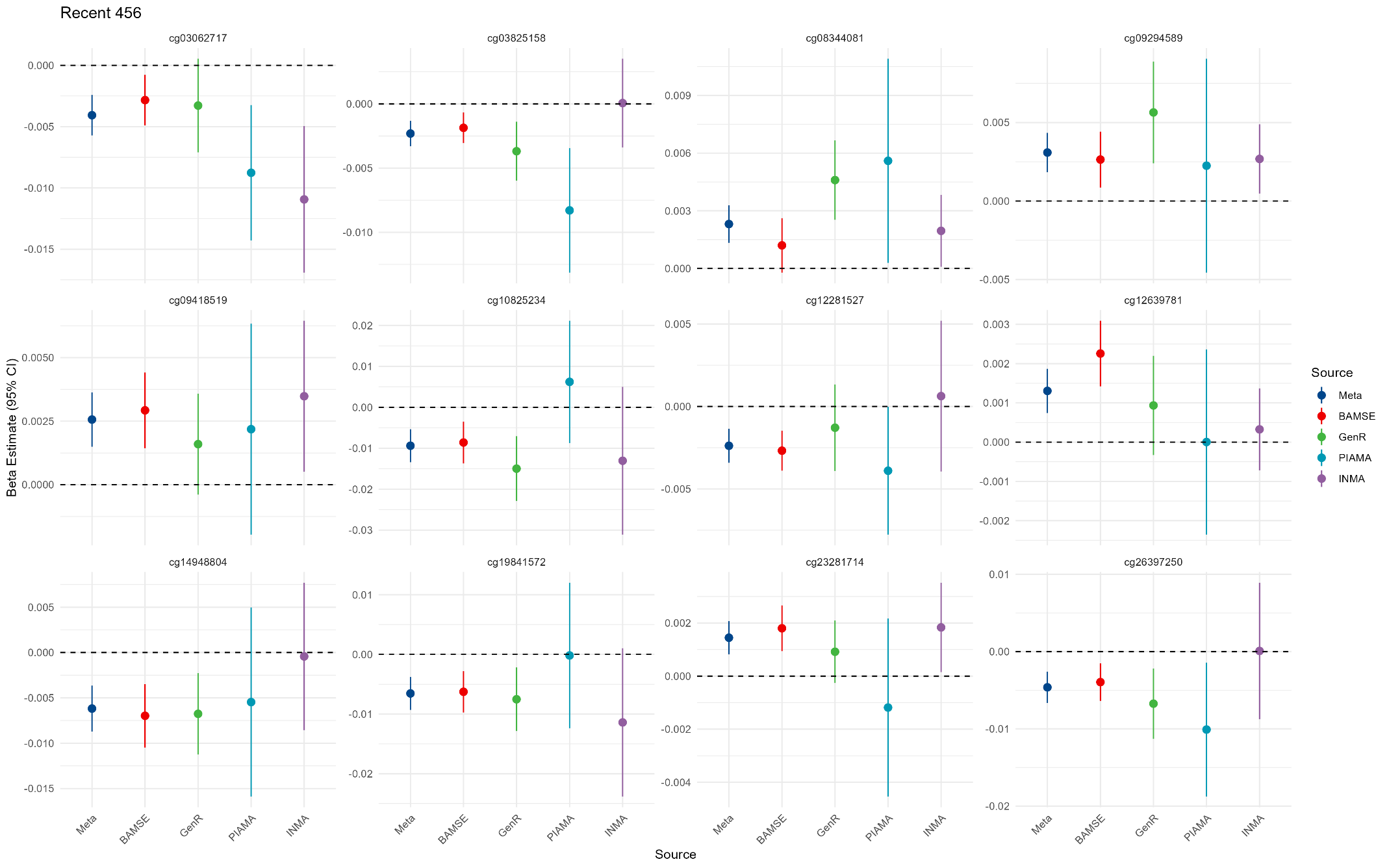


**D**


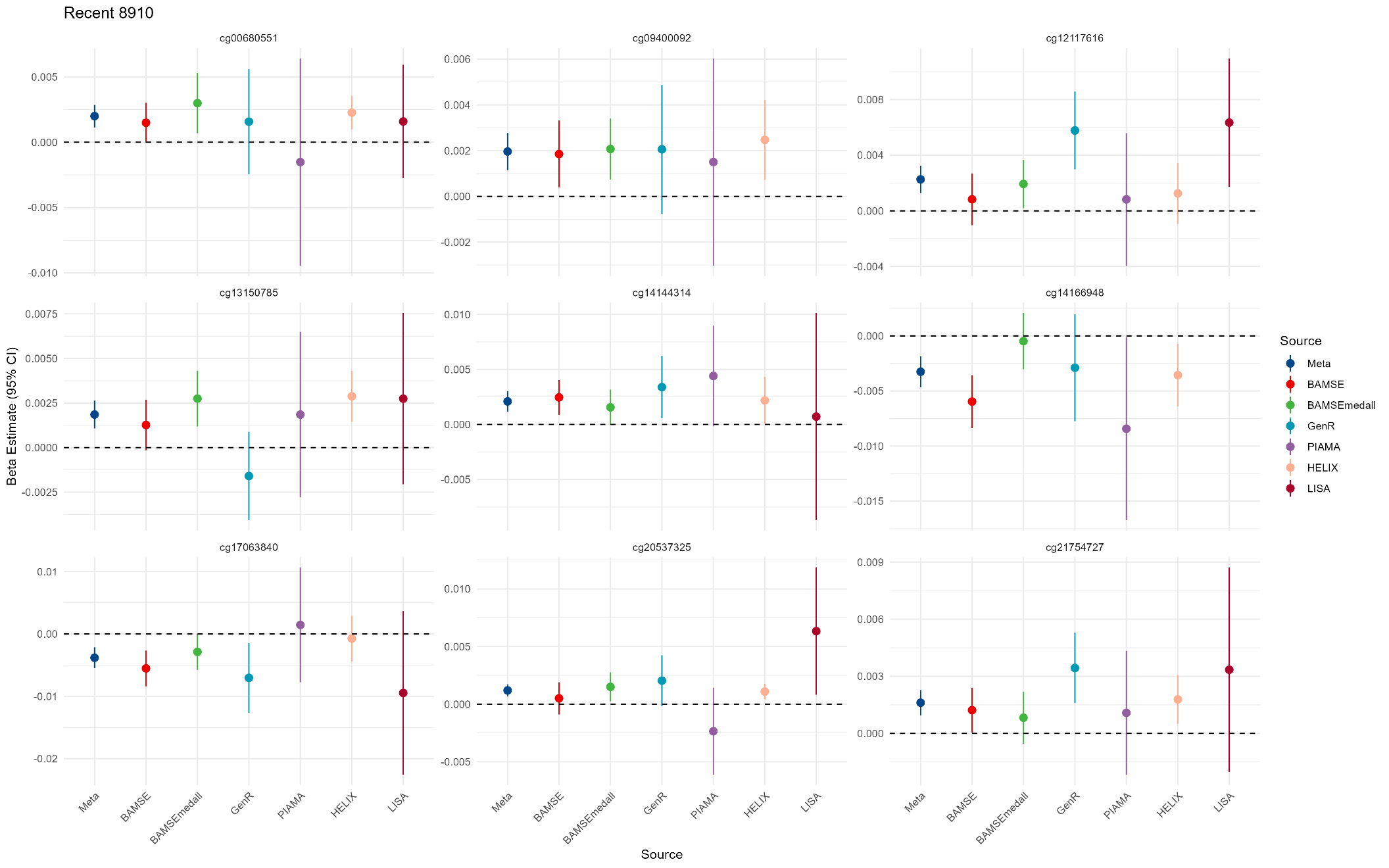


Supplemental Figure 2. Meta-analysis and cohort-specific estimates for the suggestive DMPs. (A) Infancy noise and blood DNAm at age 4-6; (B) Infancy noise exposure and blood DNAm at age 8-10 (C) Recent exposure and blood DNAm at age 4-6; (D) Recent noise exposure and blood DNAm at age 8-10.


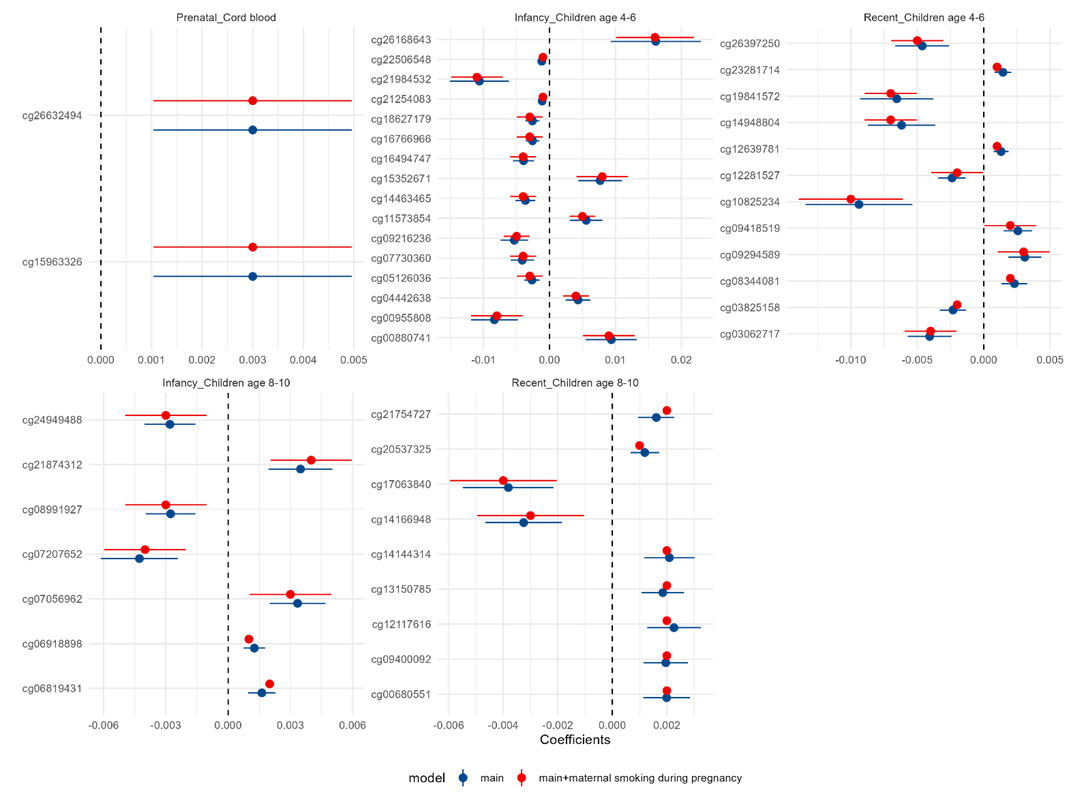


## **Supplemental Figure 3** Comparisons of the effect estimates for the suggestive DMPs in the main model and sensitivity analysis additionally adjusted for maternal smoking during pregnancy

A. Infancy noise exposure and blood DNAm at age 4-6


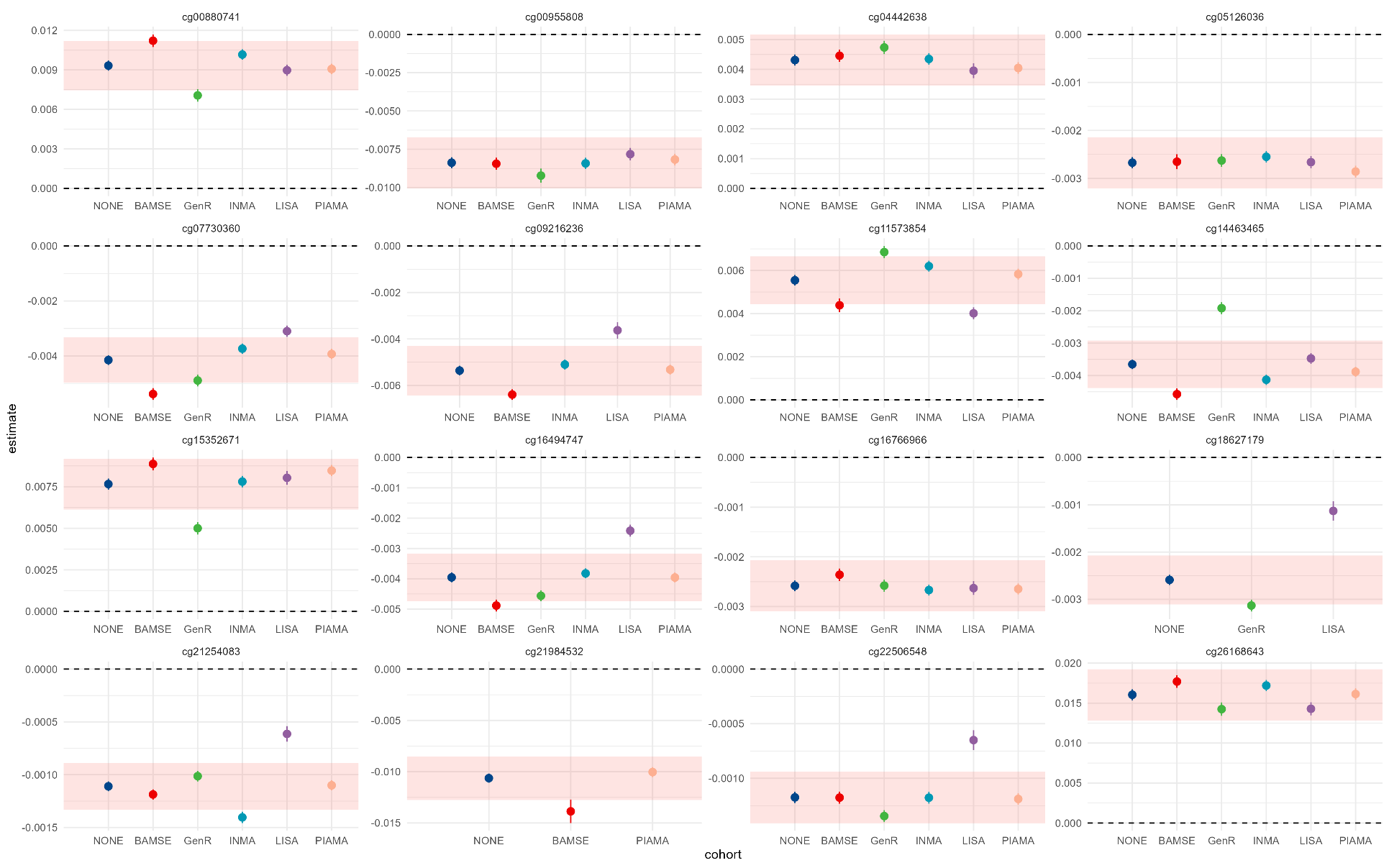


B Recent noise exposure and blood DNAm at age 4-6


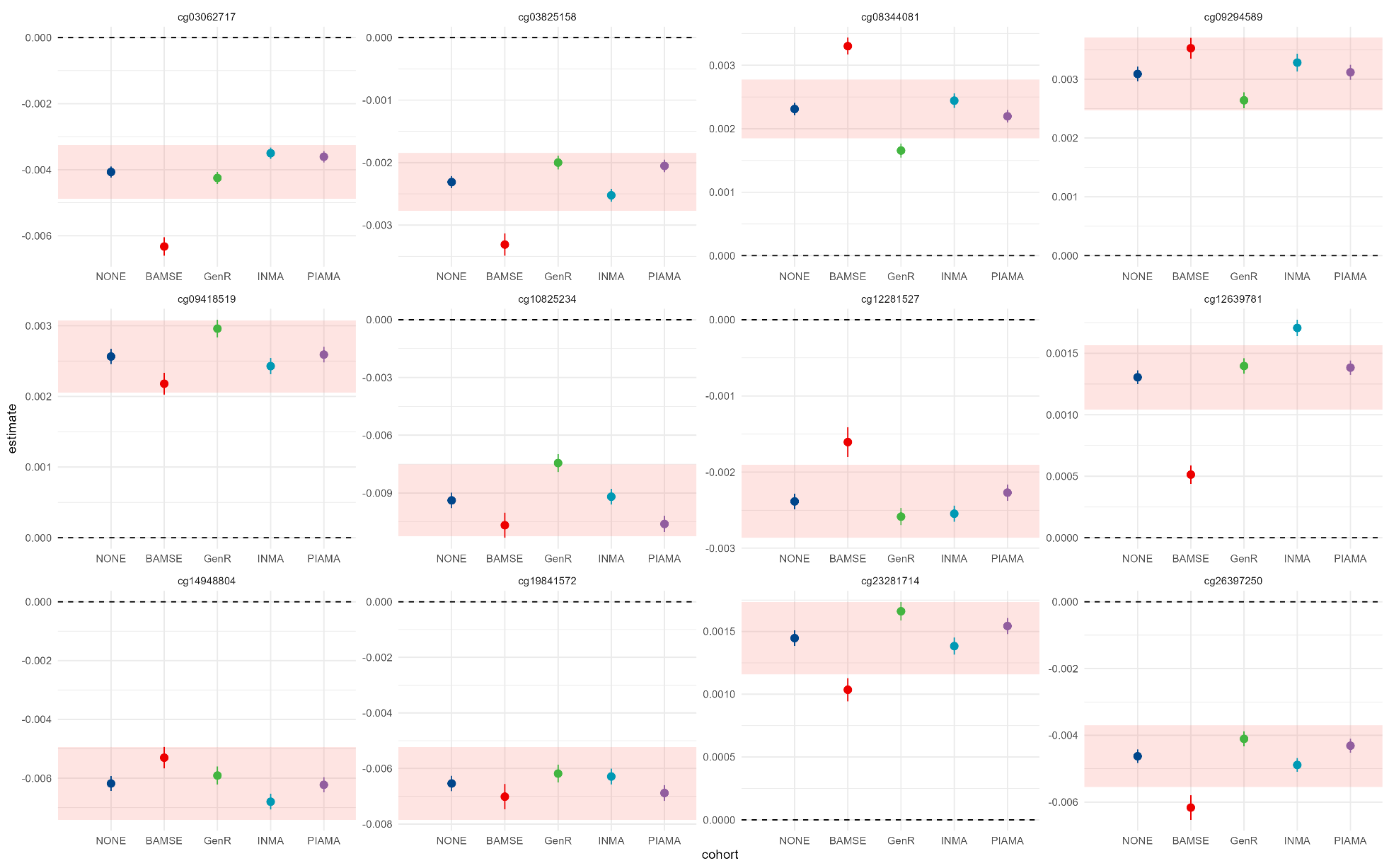


C Infancy noise exposure and blood DNAm age 8-10


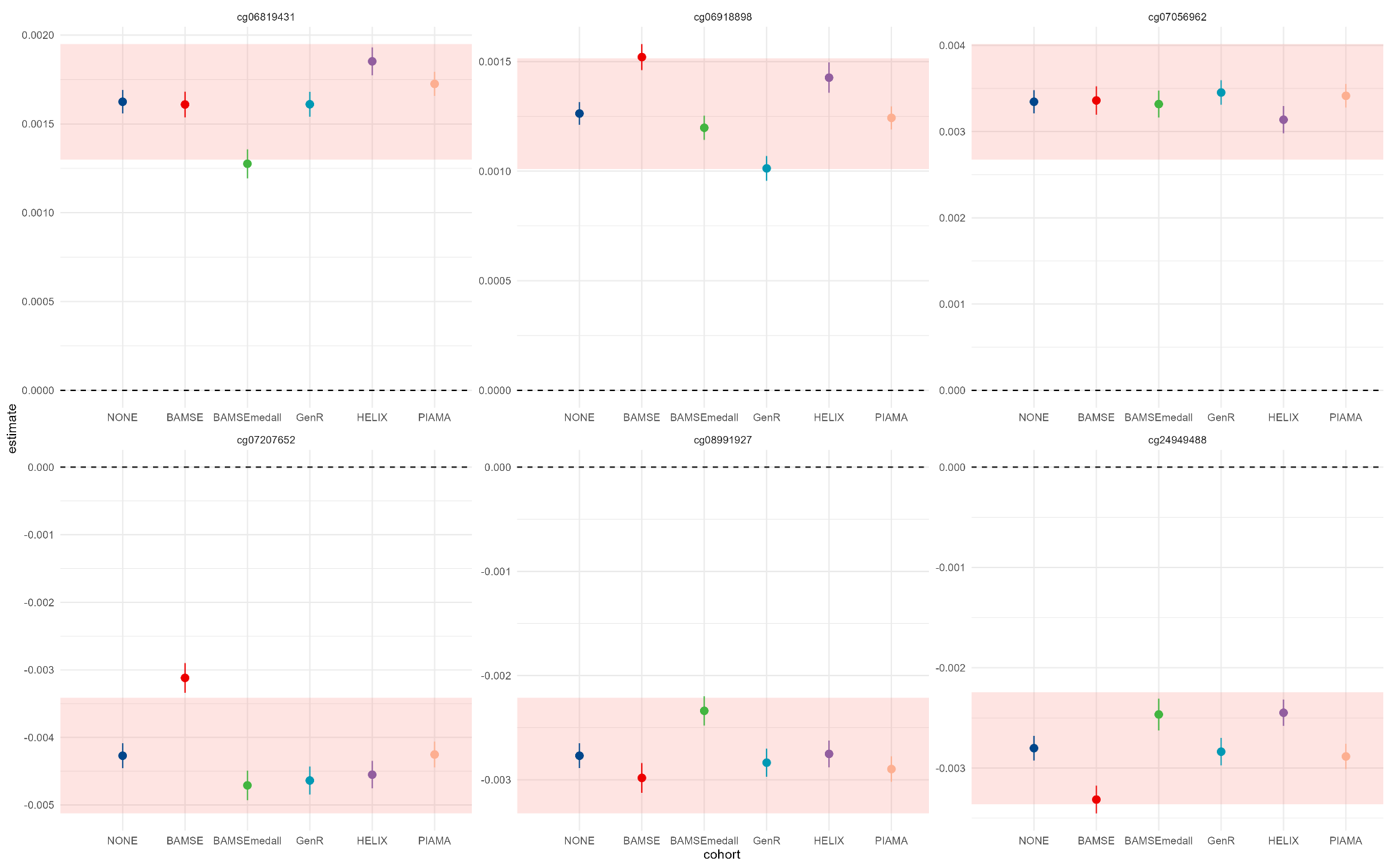


D Recent noise exposure and blood DNAm at age 8-10.


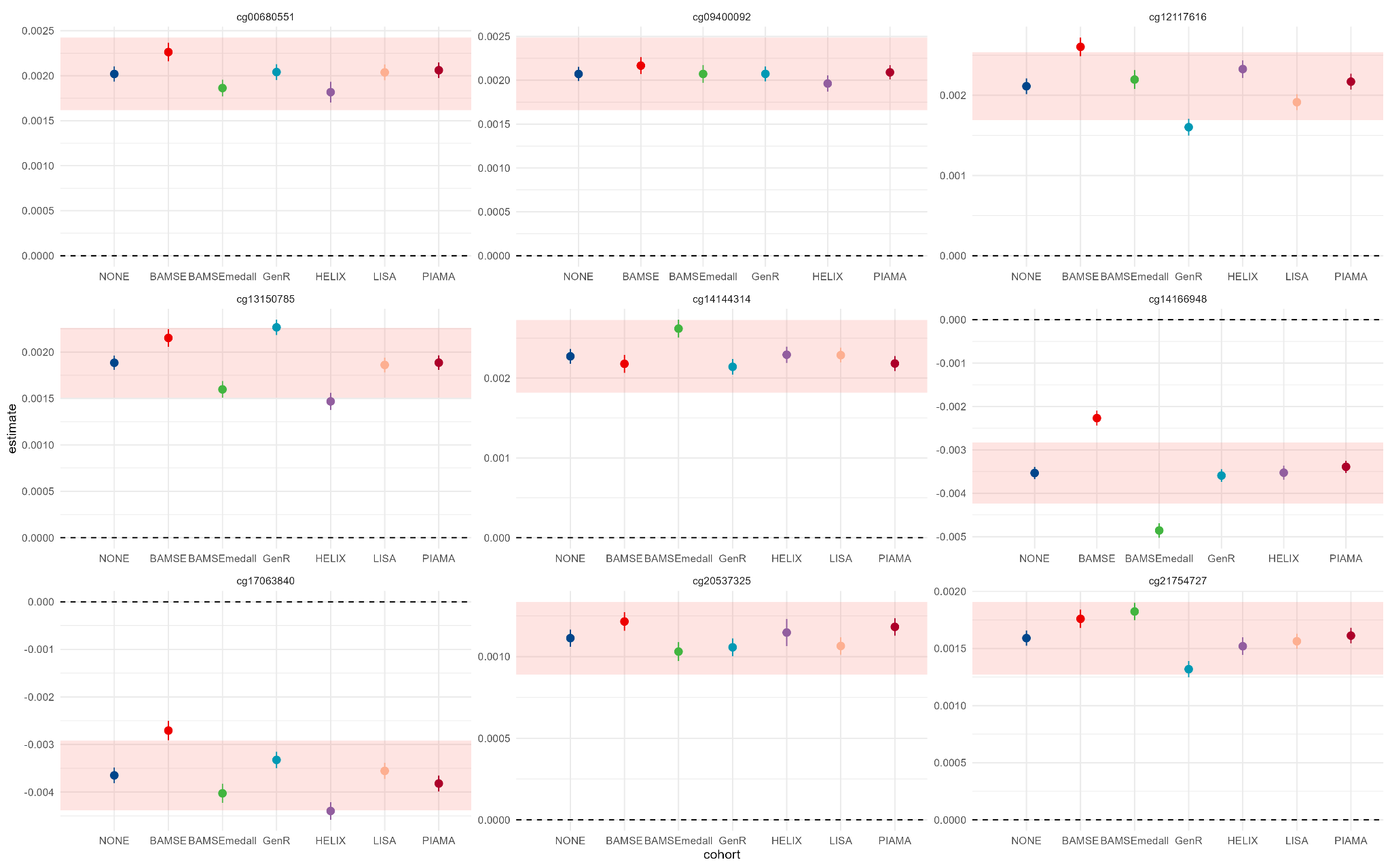


**Supplemental Figure 4 Leave-one-out meta-analysis for the suggestive DMPs in the discovery meta-analysis.** (A) Infancy noise exposure and blood DNAm at age 4-6; (B) Recent noise exposure and blood DNAm at age 4-6 (C) Infancy noise exposure and blood DNAm at age 8-10; (D) Recent noise exposure and blood DNAm at age 8-10. The pink band indicating the ±20% range of the meta-combined estimates for specific CpG sites.

A. BAMSE Epigene


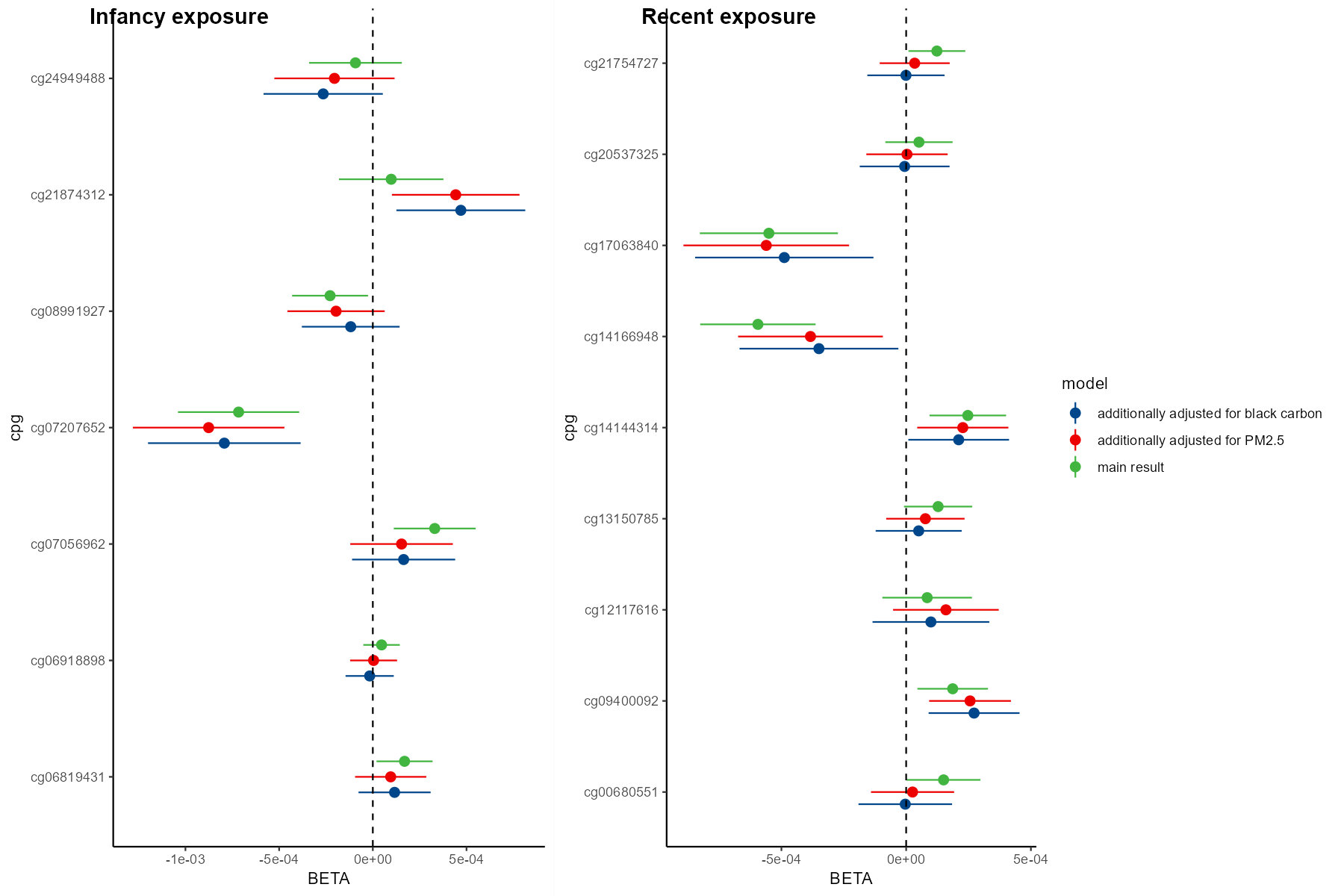


B. PIAMA


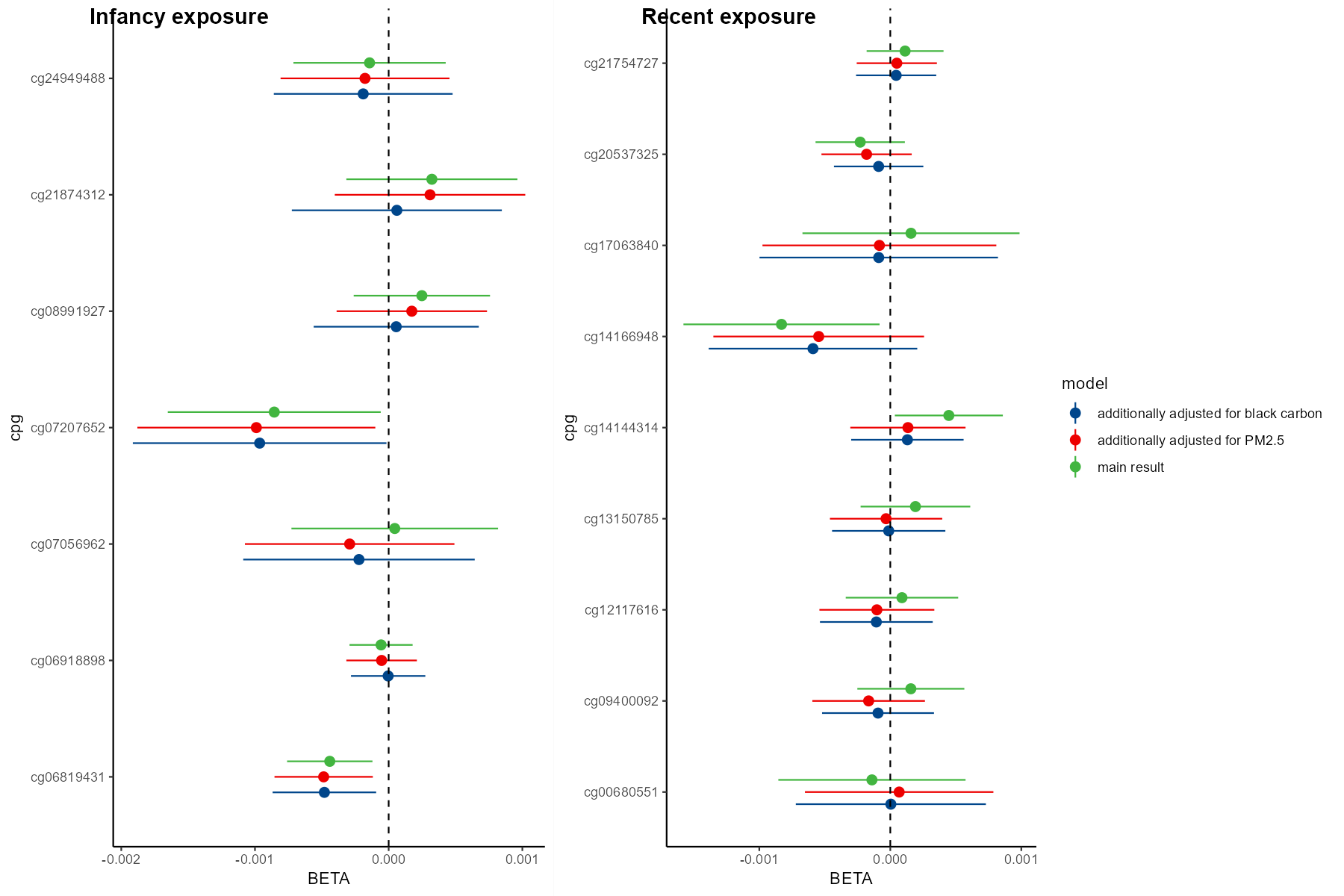


C. Generation R


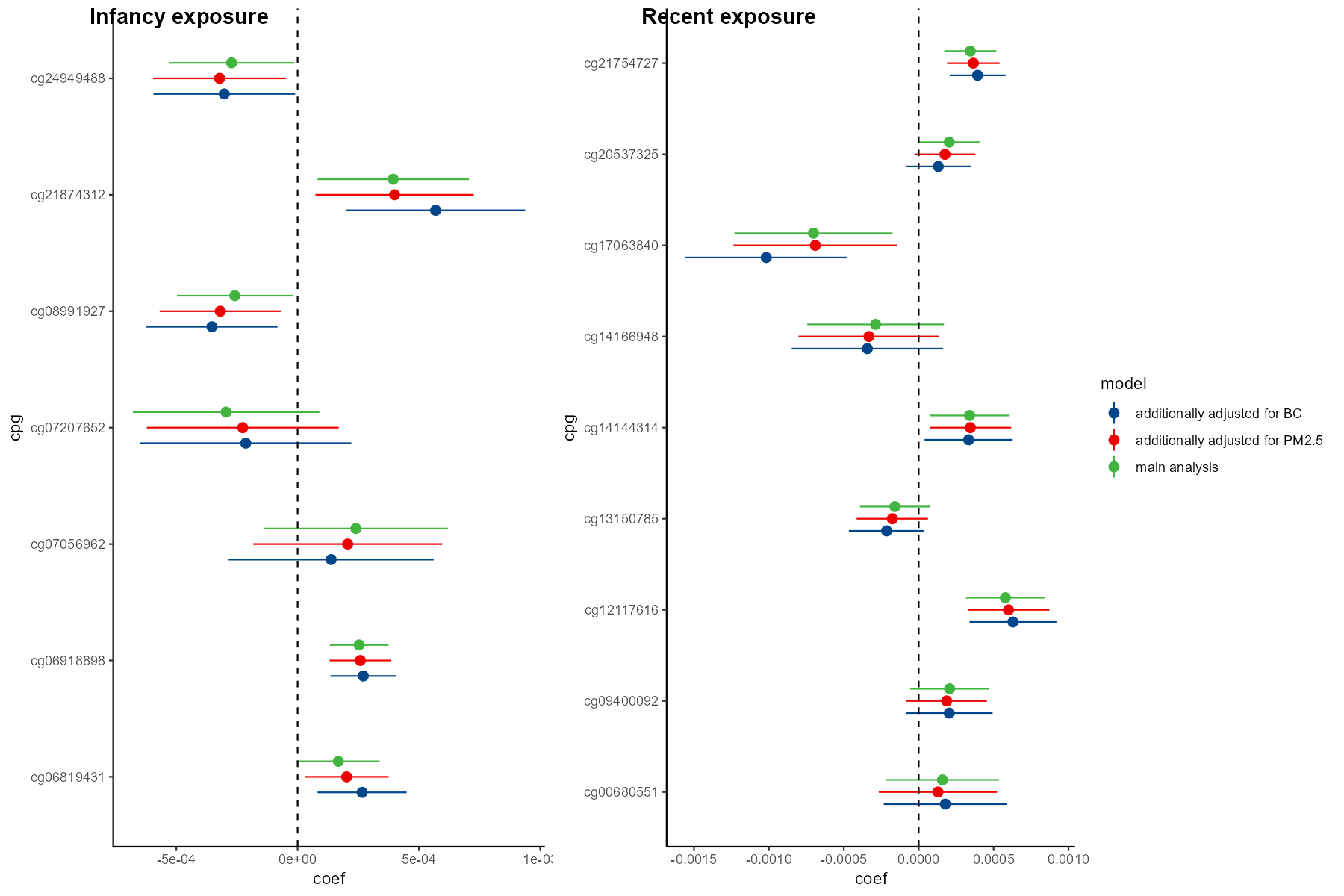


D.LISA


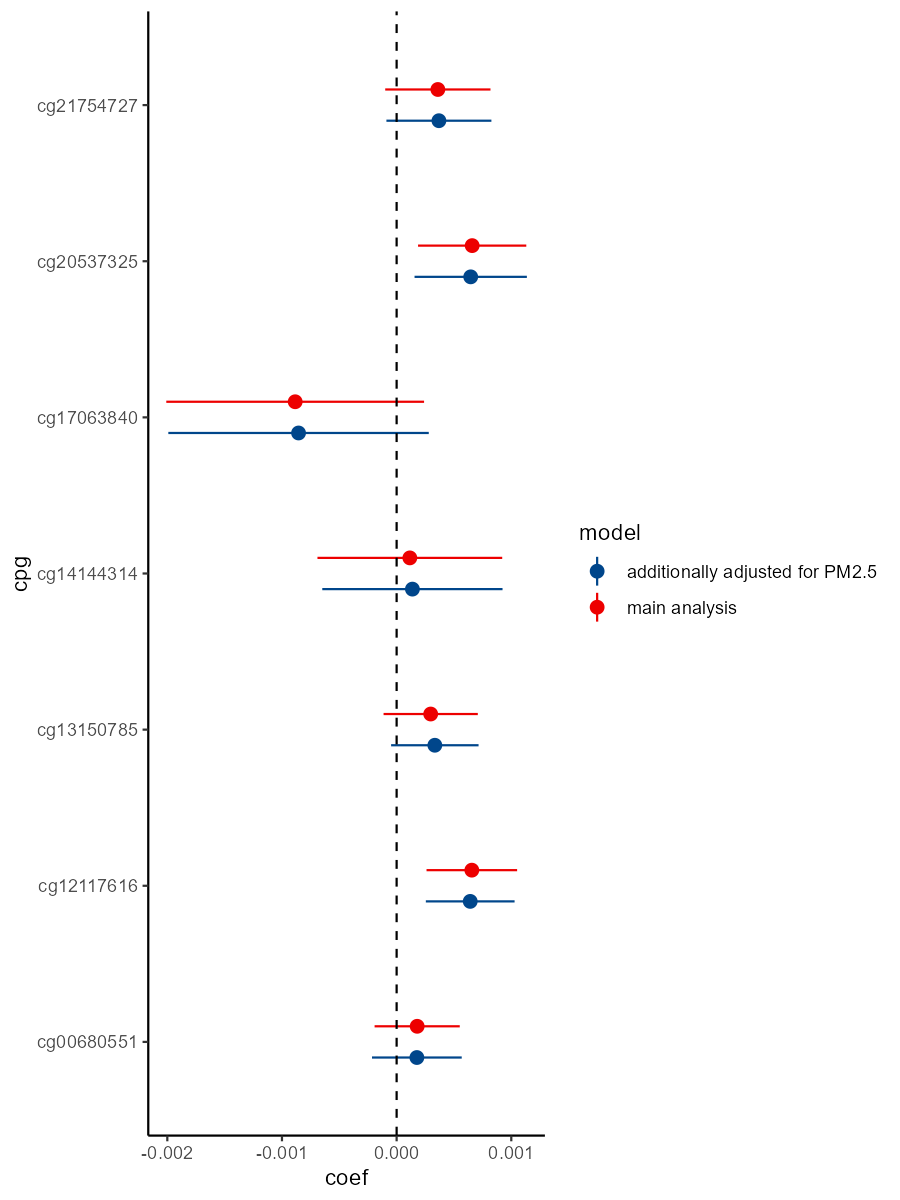


E.HELIX


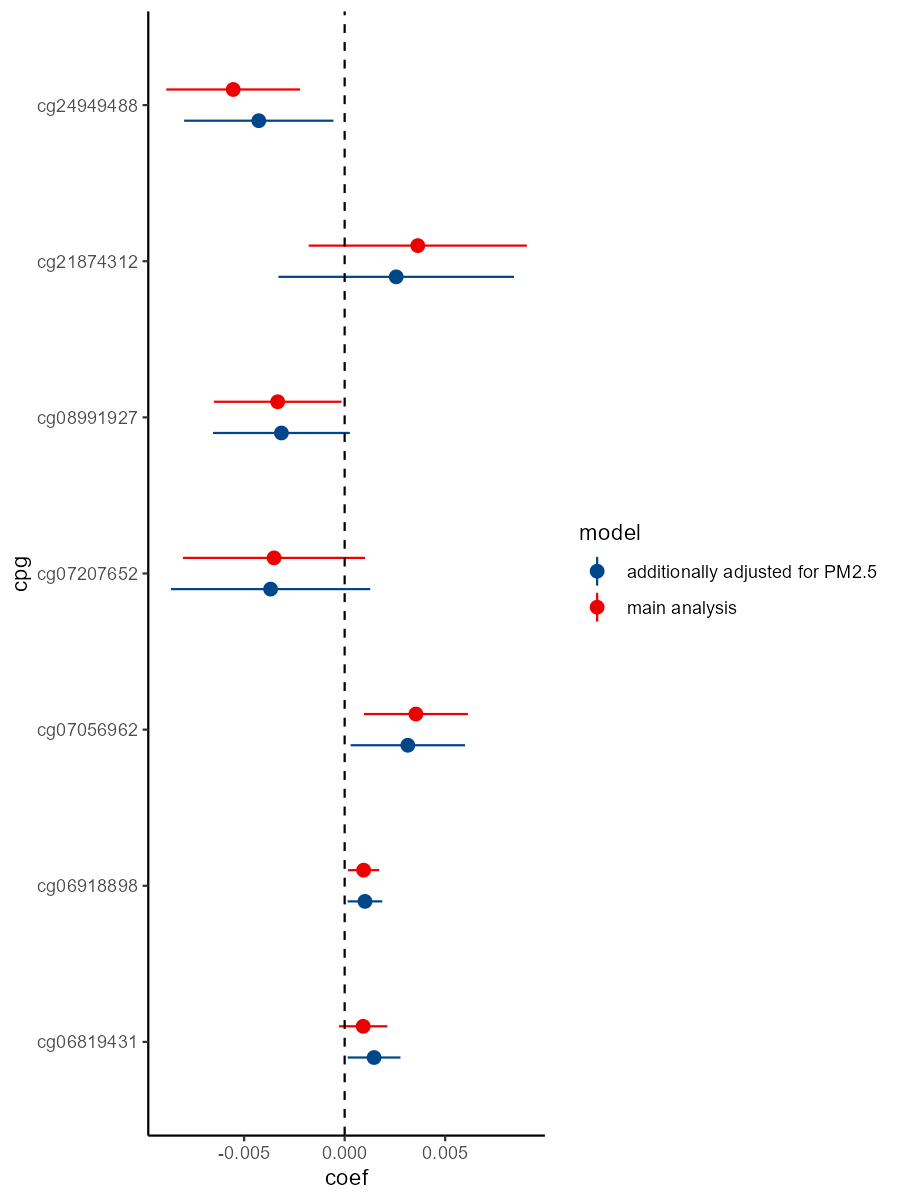


## **Supplemental Figure 5** Estimates for the suggestive DMP in children aged 8-10 in the BAMSE Epigene dataset (Panel A), PIAMA (Panel B), Generation R (panel C), LISA (panel D), HELIX (panel E) additionally adjusted for PM2.5 and black carbon. In LISA and HELIX only adjusting for PM_2.5_ are conducted due to lack of exposure data.

**a**


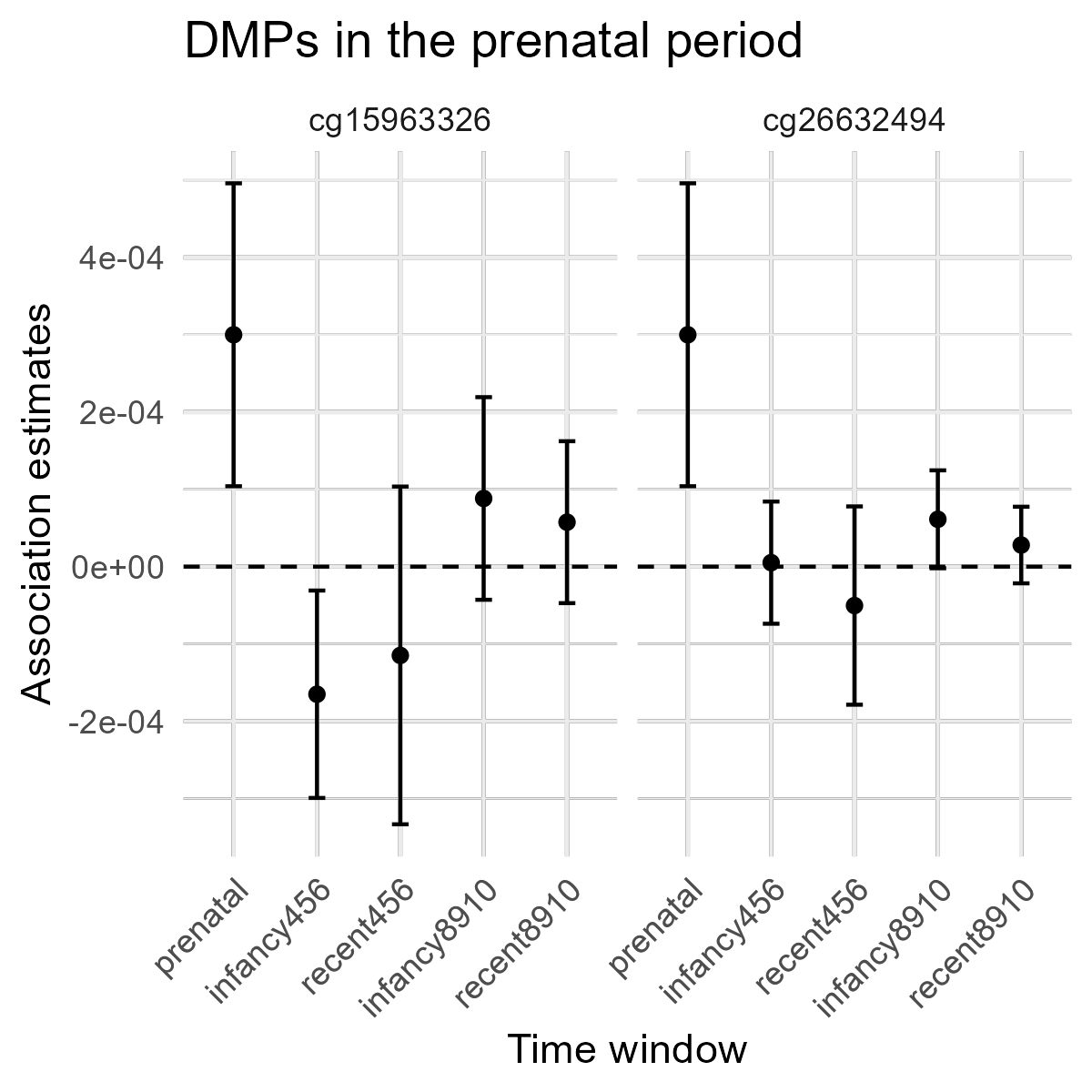


**b**


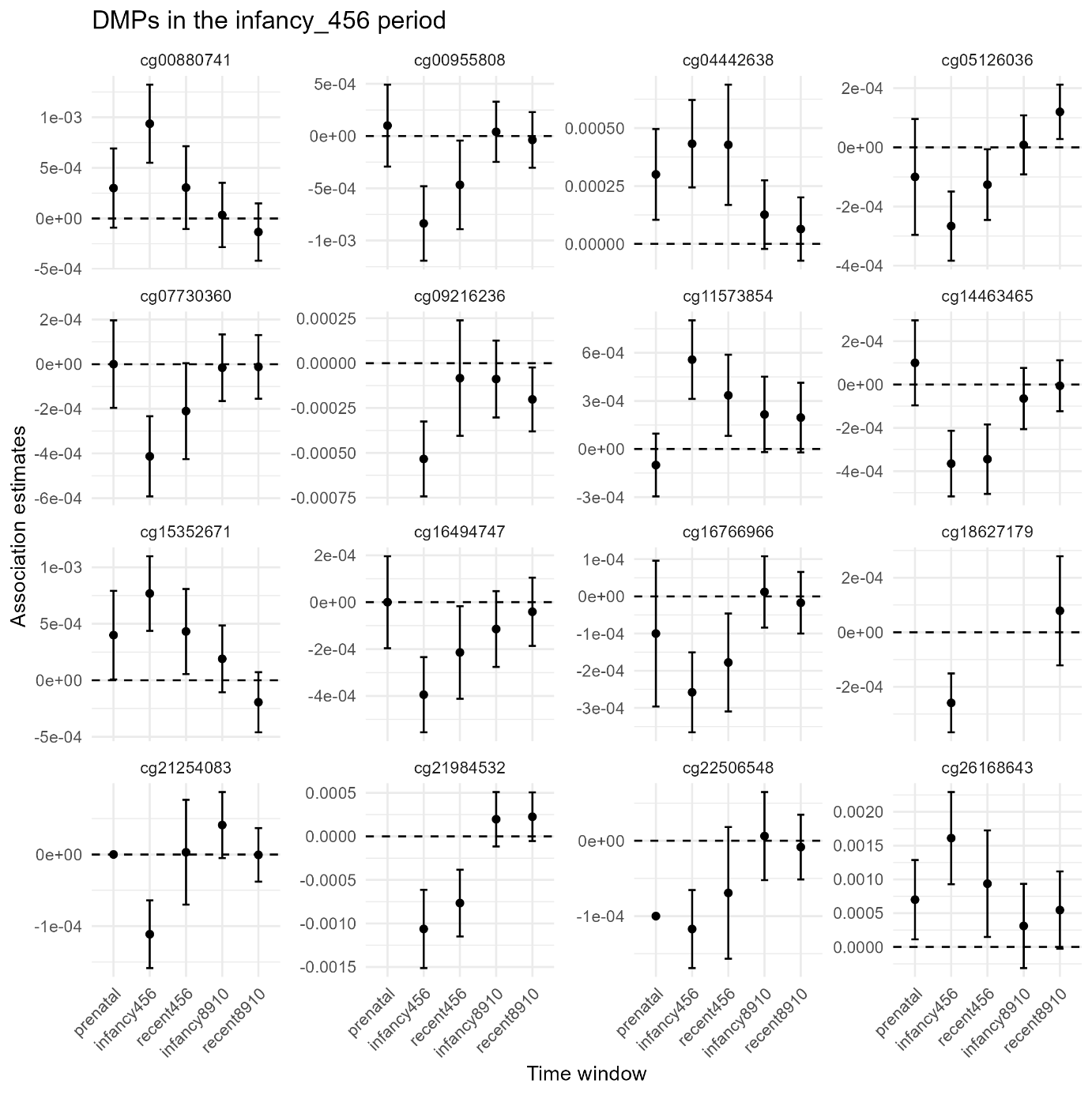


**c**


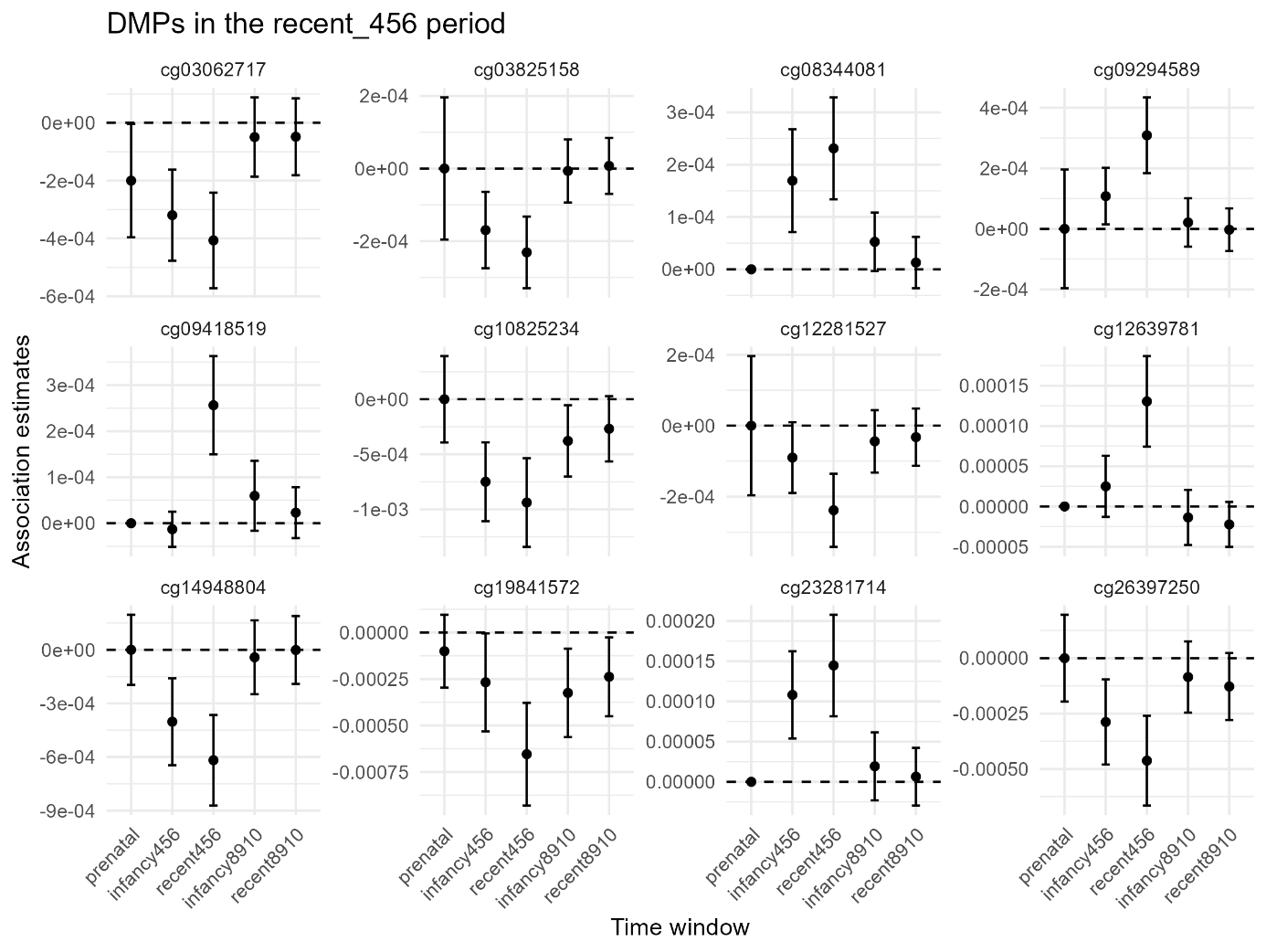


**d**


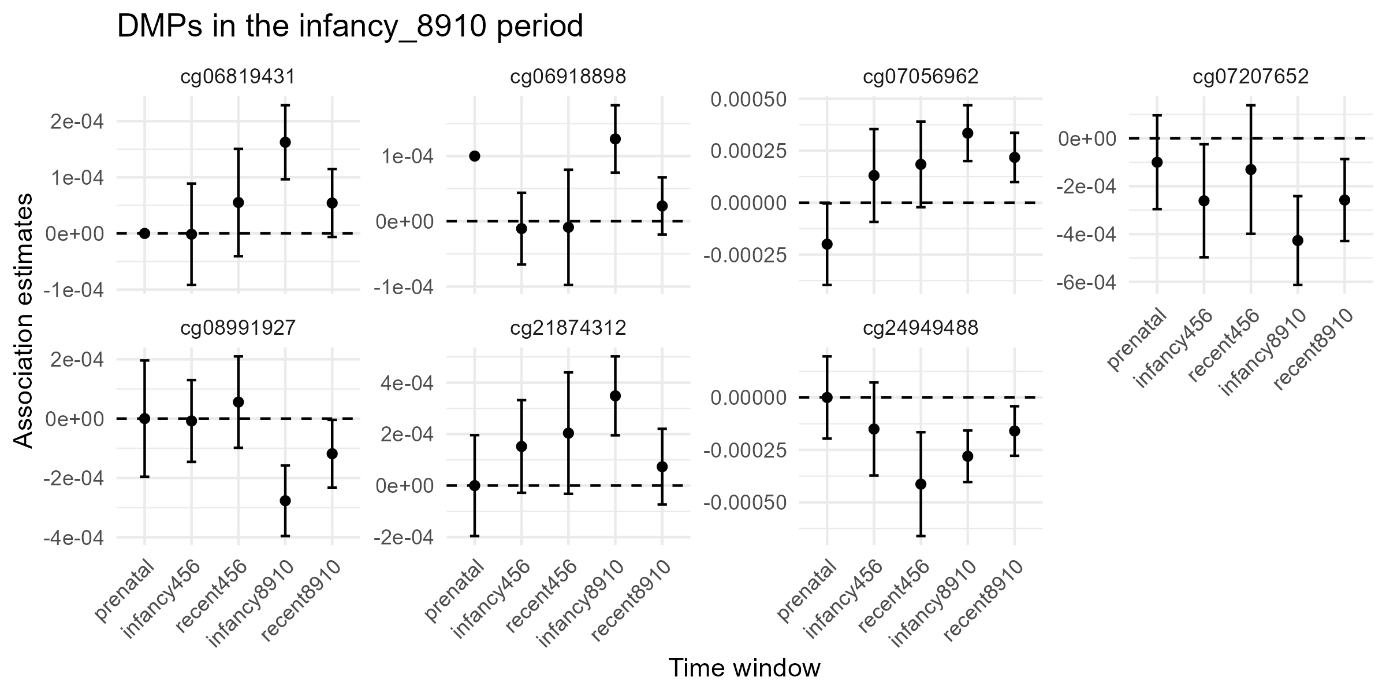


**e**


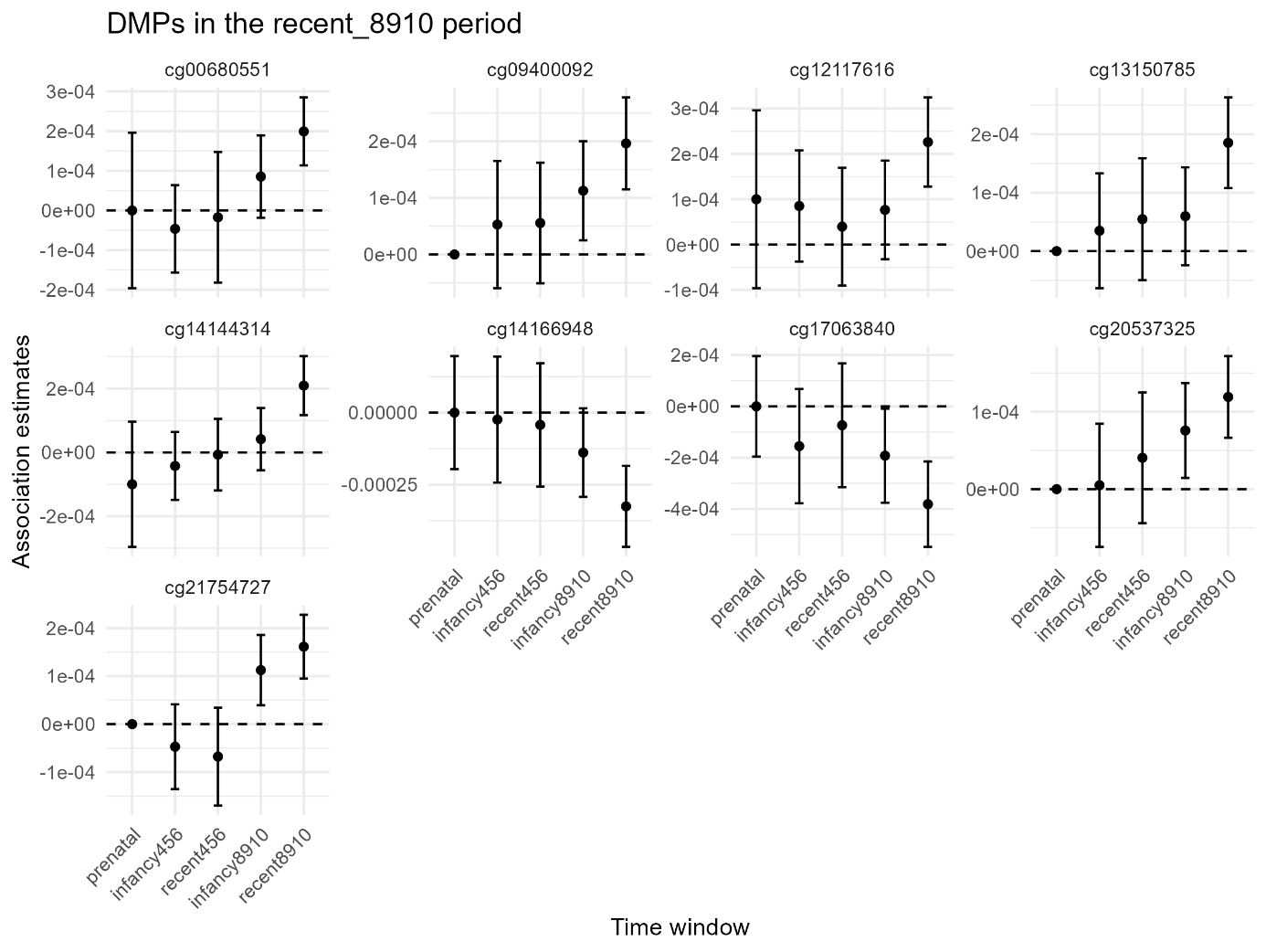


**Supplemental Figure 6 Cross look up of the suggestive DMPs in all time windows**: a) prenatal, b) infancy 4-6 years, c) recent 4-6 years, d) infancy 8-10 years, e) recent 8-10 years


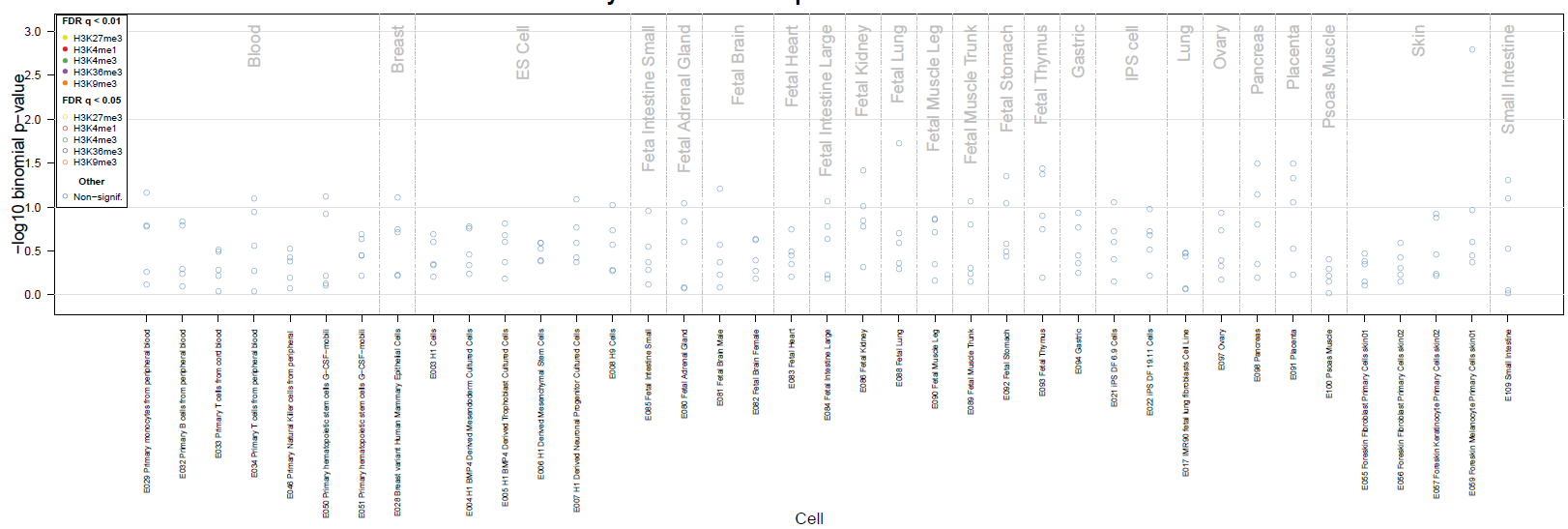


## **Supplemental Figure 7** Tissue- or cell-specific signals for the suggestive significant DMPs from the eFORGE database


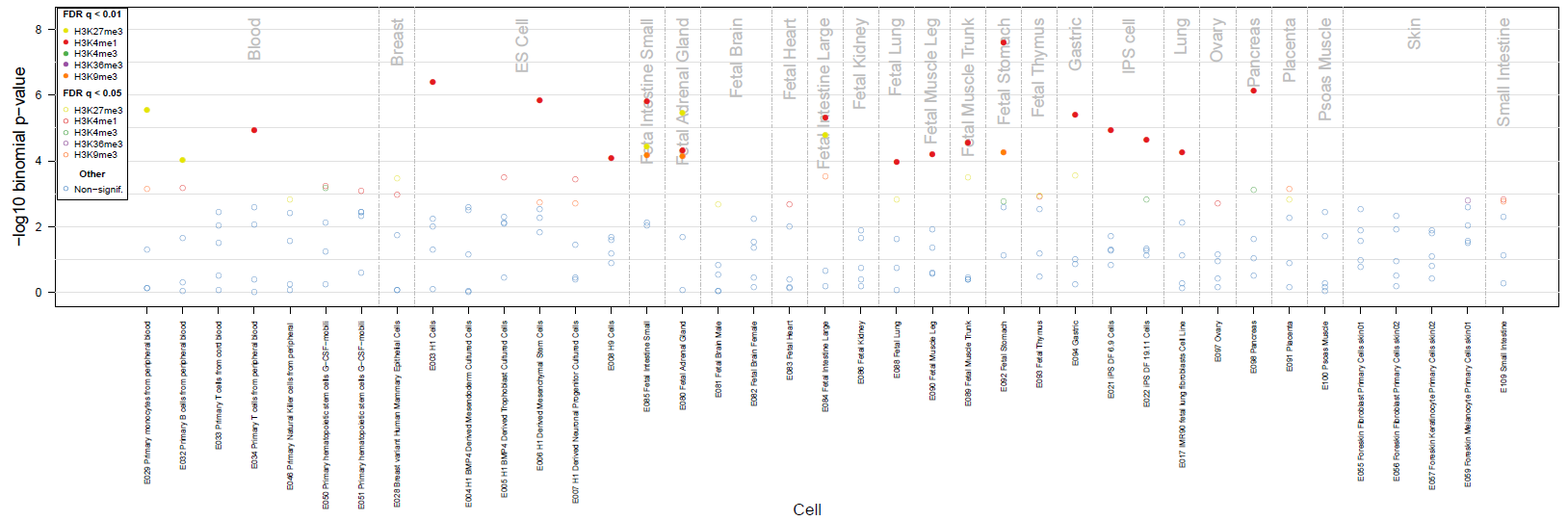


## **Supplemental Figure 8** Tissue- or cell-specific signals for all the CpGs within the significant DMRs from the eFORGE database
